# Tribbles-1 Expression and Its Function to Control Inflammatory Cytokines, Including Interleukin-8 Levels are Regulated by miRNAs in Macrophages and Prostate Cancer Cells

**DOI:** 10.3389/fimmu.2020.574046

**Published:** 2020-11-27

**Authors:** Chiara Niespolo, Jessica M. Johnston, Sumeet R. Deshmukh, Swapna Satam, Ziyanda Shologu, Oscar Villacanas, Ian M. Sudbery, Heather L. Wilson, Endre Kiss-Toth

**Affiliations:** ^1^Department of Infection, Immunity and Cardiovascular Disease, Medical School, University of Sheffield, Sheffield, United Kingdom; ^2^Department of Molecular Biology and Biotechnology, Sheffield Institute for Nucleic Acids, University of Sheffield, Sheffield, United Kingdom; ^3^Institute for Diabetes and Cancer IDC, Helmholtz Center, Munich, Germany; ^4^Health Sciences Research Centre, University of Beira Interior, Covilhã, Portugal; ^5^Intelligent Pharma, Barcelona, Spain

**Keywords:** miRNA, Tribbles, macrophages, prostate cancer, inflammation

## Abstract

The pseudokinase *TRIB1* controls cell function in a range of contexts, by regulating MAP kinase activation and mediating protein degradation *via* the COP1 ubiquitin ligase. *TRIB1* regulates polarization of macrophages and dysregulated Trib1 expression in murine models has been shown to alter atherosclerosis burden and adipose homeostasis. Recently, *TRIB1* has also been implicated in the pathogenesis of prostate cancer, where it is often overexpressed, even in the absence of genetic amplification. Well described TRIB1 effectors include MAP kinases and C/EBP transcription factors, both in immune cells and in carcinogenesis. However, the mechanisms that regulate *TRIB1* itself remain elusive. Here, we show that the long and conserved 3’untranslated region (3’UTR) of *TRIB1* is targeted by miRNAs in macrophage and prostate cancer models. By using a systematic *in silico* analysis, we identified multiple “high confidence” miRNAs potentially binding to the 3’UTR of *TRIB1* and report that miR-101-3p and miR-132-3p are direct regulators of *TRIB1* expression and function. Binding of miR-101-3p and miR-132-3p to the 3’UTR of *TRIB1* mRNA leads to an increased transcription and secretion of interleukin-8. Our data demonstrate that modulation of *TRIB1* by miRNAs alters the inflammatory profile of both human macrophages and prostate cancer cells.

## Introduction

Tribbles-1 (*TRIB1*) is an evolutionary conserved pseudokinase that in mammals controls a wide range of interacting signaling pathways, such as mitogen activated protein kinases and the PI3-kinase pathway. Its expression is detectable in most tissues; it is highest in the bone marrow and in the thyroid gland (The Human Protein Atlas, https://www.proteinatlas.org). Structurally, the TRIB1 protein contains an N-terminal PEST region, a pseudokinase domain and a C-terminal COP1-binding region. By lacking a functional adenosine 5’-triphosphate (ATP) binding site, TRIB1 is a catalytically inactive enzyme (1). However, through the pseudokinase domain, TRIB1 acts as adapter or scaffold facilitating assembly with other proteins (2–4). *TRIB1* is an established oncogene in acute myeloid leukemia (5, 6) and has been shown to play a pivotal role in the differentiation of anti-inflammatory M2-macrophages in the pathogenesis of early atherosclerosis and during adipose tissue inflammation (7–9). Additionally, *TRIB1* has been reported to be overexpressed in prostate cancer, where it controls the expression of endoplasmic reticulum chaperone proteins and induces M2-macrophage differentiation (10, 11). At the transcript level, *TRIB1* is highly unstable, reported to have an mRNA half-life shorter than 1 hour (12). *TRIB1* expression is also highly variable among different cell types and tissues, suggesting it might be subject to cell type-dependent post-transcriptional regulation (13, 14). In fact, the 2kb long, 3’untraslated region (3’UTR) of *TRIB1* mRNA represents more than 50% of the entire sequence and is highly conserved among different animal species. Recently, Soubeyrand and colleagues showed that blocking transcription through actinomycin-D dramatically reduced *TRIB1* mRNA levels in HeLa cells and Aortic Smooth Muscle cells (15), while overexpression of the 3’UTR was shown to raise levels of the endogenous mRNA in HeLa cells (2). A major gene regulatory mechanism described in eukaryotes is mediated by microRNAs (miRNAs) by the process of RNA interference (RNAi). miRNAs are small non-coding RNAs that commonly bind to the 3’UTR of the target gene, and, *via* association with effector proteins, form the RNA-induced silencing complex (RISC) that ultimately leads to RNA degradation and inhibition of protein translation (16).

In the present work, we systematically analyzed the post-transcriptional regulation of *TRIB1* by miRNAs, with focus on macrophages and prostate cancer models. We show that the 3’UTR of *TRIB1* significantly reduces expression of a luciferase reporter when transiently transfected into HEK293 cells, it is enriched in miRNA-binding sites and is a direct target of multiple miRNAs. A significant number of miRNAs predicted to regulate *TRIB1*, are also silenced and/or downregulated in prostate cancer, and therefore may contribute to the elevated expression of this pseudokinase in this cancer. By directly targeting *TRIB1*, miR-101-3p and miR-132-3p control the inflammatory profile of human primary macrophages and prostate cancer cells, as exemplified by enhancing the expression and secretion of pro-inflammatory chemokine, interleukin-8.

## Materials and Methods

### Statement of Ethics

To isolate primary human blood monocytes, up to 80 ml of venous blood was taken from healthy donors. The study was approved by the University of Sheffield Ethics Committee (Reference Numbers: 031330, SMBRER310) in accordance with the Declaration of Helsinki and written informed consent was obtained from all volunteers.

### Animal Experiments

Mice were handled in accordance with UK legislation (1986) Animals (Scientific Procedures) Act. Mouse experiments were approved by the University of Sheffield Project Review Committee and carried out under a UK Home Office Project License (70/7992). Myeloid specific *Trib1* conditional knockout (KO) mice were generated by crossing *Trib1 fl/fl* mice that contain flanking loxP sites around the second exon of *Trib1* and crossing them with *Lyz2Cre* recombinase transgenic mice (www.jax.org/strain/004781). The resulting mice; *Trib1 fl/fl* x *Lyz2Cre* (*Trib1^m^*^KO^) have all but the first 120 amino acids of TRIB1 excised. Myeloid specific *Trib1* over-expressor transgenic mice were generated by crossing *Rosa26.Trib1* mice with *Lyz2Cre* recombinase transgenic mice as described above. The resulting mice; Rosa26.Trib1 x Lyz2Cre (*Trib1^m^*^Tg^) over-express *Trib1* transgene by ~2.5 fold as previously described by Johnston et al. (9). Wild- type litter mates (*Trib1^m^*^WT^) were used as controls. All mice used were congenic on a C57BL/6J background and were housed in a controlled environment with a 12-h light/dark cycle, at 22°C in Optimice individually ventilated cages (Animal Care Systems) and given free access to a standard chow diet (#2918; Harlan Teklad) and water. Mice were sacrificed humanely by cervical dislocation at 12–13 weeks of age.

### Isolation of Human Monocyte-Derived Macrophages (MDMs)

Peripheral blood mononuclear cells (PBMCs) were isolated by Ficoll–Paque Plus (GE Healthcare) density centrifugation and CD14+ monocytes were selected by positive magnetic separation using CD14 microbeads (Miltenyi Biotec). Monocytes were then cultured in complete media for seven days: RPMI-1640 (Gibco), 10% (v/v) low-endotoxin heat-inactivated FBS (PanBiotech), 1% (v/v) L-glutamine (Gibco) and 1% (v/v) penicillin/streptomycin (Gibco). Recombinant human (rh) M-CSF (100 ng/ml, Peprotech/Immunotools) was added to the media to allow the differentiation of monocytes into macrophages (MDMs). After 7 days of differentiation, MDMs were polarized into pro- (M1) and anti-(M2a) inflammatory macrophages for 24 h, using 20 ng/ml IFN-γ (Human, Peprotech) and 100 ng/ml *E. coli* lipopolysaccharide (Serotype R515 TLR grade TM, Enzo Life Sciences) and 20 ng/ml IL–4 (Human, Peprotech), respectively.

### Established Cell Lines

HEK293T cells were obtained from ATCC^®^ (UK) and grown in Dulbecco’s modified Eagle’s medium (DMEM, Gibco) supplemented with 10% (v/v) low-endotoxin heat-inactivated FBS (PanBiotech) and 1% (v/v) penicillin/streptomycin (Gibco). Immortalized bone marrow-derived macrophages (iBMDMs) were kindly provided by D. Brough (University of Manchester) and generated as described by Hornung et al. (17), were grown in DMEM further supplemented with 1% (v/v) sodium pyruvate (Gibco) and 1% (v/v) sodium bicarbonate (Gibco). HEK293T cells and iBMDMs were grown in T75 flasks passaged every 2–3 days in a 1:10 ratio, using trypsin/EDTA (Lonza) and cell scrapers (Biofil), respectively. Prostate cell lines used were PC3 and LNCAP as cancer cells and PWR1E and RWPE1 as normal prostatic epithelial cell controls. PC3 were grown in complete DMEM (Gibco) and LNCAP in RPMI-1640 plus 10% (v/v) low-endotoxin heat-inactivated FBS (PanBiotech), 1% (v/v) penicillin/streptomycin (Gibco) and 1% (v/v) L-glutamine (Lonza). PWR1E and RWPE1 were cultured in Keratinocyte Serum Free Media (K-SFM, Gibco) supplied with 1% (v/v) penicillin/streptomycin, 0.05 mg/ml bovine pituitary extract (BPE) and 5 ng/ml human recombinant epidermal growth factor (EGF), both provided with the K-SFM kit.

### miRNA-Target Prediction Analysis

miRNA-target prediction analysis was carried out using seven different algorithms (miRanda 2010 (18), TargetScan v.7.2 (19), miRDB v.6 (20), StarBase 2019-2020 (21), miRwalk v.2 (22), Tarbase v.8 (23), and microT-CDS v.5 (24). All tools were used online, except miRanda which was downloaded and used locally as independent software, employing the default parameters. miRbase v.22 (25, 26) was used to download the list of “high-confidence” miRNAs, based on deep sequencing data. The total number of miRNAs predicted to target the 3’UTR of *TRIB1* by each tool were imported in mySQL Database (pgAdmin v.4) and filtered using structural queries (for example “intersect” function to intersect tables and find “common” elements). We then selected only miRNAs predicted by at least 3 different tools. To identify miRNAs dysregulated in prostate cancer, we used the microRNA Cancer Association Database miRCancer, which is based on published literature [http://mircancer.ecu.edu/ (27)].

### Transient Transfection

Small RNA-based transfection was carried out using Viromer Green for primary cells and Viromer Blue for established cell lines (Lipocalyx), while DNA-based transfection was performed using Lipofectamine 3000 (Invitrogen). Simultaneous transfection of RNA and DNA was achieved using Dharmafect DUO (Horizon Discovery). *TRIB1* siRNA, miRNA mimics, inhibitors, and negative controls were purchased from Horizon Discovery (sequences are listed in **Supplementary Table 1**); miR-101/*TRIB1* Target site blocker (TSB) and negative control were obtained from Qiagen. TSB are antisense oligonucleotides designed to bind to a specific region of a miRNA-target gene, thus masking it from the miRNA. However, the TSB does not lead to the activation of the RISC complex; it only prevents the endogenous miRNA from binding its target (for further technical details, see: https://www.qiagen.com/gb/products/discovery-and-translational-research/functional-and-cell-analysis/mirna-functional-analysis/mircury-lna-mirna-power-target-site-blockers/mircury-lna-mirna-power-target-site-blockers/#productdetails). Each transfection experiment was performed independently. Transfection time and RNA/DNA concentrations are specified in each figure legend.

### Immunofluorescence Staining

To assess Kupffer cell phenotype, FFPE liver tissue from mixed gender mice was stained with F4/80 and phenotypic markers YM1 (M2/alternatively activated) and IRF5 (M1/classically activated). Adipose tissue macrophages were stained with F4/80 and YM1 only. Sections were de-waxed, rehydrated and endogenous peroxidases were blocked by incubation in 3% (v/v) hydrogen peroxidase for 10 min at room temperature followed by enzyme induced (trypsin) antigen retrieval (A. Menarini Diagnostics, UK) and permeabilized with 0.1% (v/v) Triton X-100 for 15 min. Tissues were incubated with rat anti-F4/80 and then with a secondary biotinylated rabbit anti-rat followed by Streptavidin-PE. Sections were then re-blocked in 5% (v/v) donkey serum and incubated with either mouse anti-mouse IRF5 or rabbit anti-mouse YM1 followed by either incubation with donkey anti-mouse or donkey anti-rabbit NL493. Details of antibodies, dilutions and incubation time can be found in **Supplementary Table 2**. Tissues were then mounted with ProLong^®^ Gold antifade mountant with DAPI (Molecular Probes). Fluorescent images were captured using an inverted wide-field fluorescence microscope (Leica AF6000) and analyzed by Image J64 (v1.51). Only cells that were F4/80+ were included in the analysis to ensure macrophage specificity. Relative IRF5 and YM1 staining is normalized to levels of F4/80+ cells and are representative of at least three fields of view. Specificity of immunofluorescence staining was ascertained by using isotype controls as exemplified in **Supplementary Figure 1**.

### Real-Time Quantitative PCR

Total RNA isolation was performed by using the miRNeasy Mini Kit (Qiagen) according to manufacturer instructions. The kit enables the purification of total RNA, including small RNAs and is based on silica-spin columns for optimal RNA binding. RNA concentration and purity were assessed by NanoDrop™ Spectrophotometer (260/280 ratio = 1.8/2.0; 260/230 ratio = 1.8/2.0). cDNA was synthesized using iScript cDNA Synthesis Kit (Biorad) for mRNA and miRCURY LNA RT Kit for microRNAs (Qiagen) following manufacturer instructions. Real-time quantitative PCR (RT-qPCR) was performed using SYBR Green master mix (Primer Design) for the mRNA analysis; the miRCURY LNA miRNA PCR Assay kit (Qiagen) was used for microRNAs amplification. Primers were designed and checked for specificity using BLAST Primer Design Tool (https://www.ncbi.nlm.nih.gov/tools/primer-blast) and purchased from Sigma-Aldrich; microRNA-specific primer mix were obtained from Qiagen. Results were analyzed upon a CFX384 C1000 Touch Thermal Cycler (Biorad), using the 2(-Delta Ct) analysis method. Each biological sample analyzed represents the average value of 3 technical replicates. Primer sequences are listed in **Supplementary Table 3**.

### Western Blotting

Proteins were extracted from cell culture plates by homogenizing in RIPA buffer (Sigma-Aldrich), containing protease inhibitors (Sigma-Aldrich). The lysates were centrifuged at 4°C for 20 min at 14,000 g and the liquid upper phase was collected. Five or ten (depending on the specific experiment) micrograms of total protein lysate were then loaded onto a 4% to 12% NuPAGE Bis-Tris Gel (Invitrogen) and transferred onto Immobilon PVDF Membrane (Merck). Primary and secondary antibodies used are listed in **Supplementary Table 2**.

### Enzyme-Linked Immunosorbent Assay (ELISA)

IL-8 ELISA was carried out using DuoSet ELISA kit (R&D) following manufacturer’s instructions. The samples were supernatants collected after transient transfection of human MDMs.

### Molecular Cloning and Site-Directed Mutagenesis

Molecular cloning was carried out to generate reporter and expression plasmids by using pENTR/D-TOPO Cloning Kit (Invitrogen). High-efficiency transformation of bacteria was performed using 5-alpha competent *E. coli* (New England’s Labs). Multiple nucleotides deletions were generated through a site-directed mutagenesis kit according to manufacturer instructions (Agilent). Mutagenic primer sequences are specified in **Supplementary Table 4**.

### Dual Luciferase Reporter Assay

HEK293 cells were transfected with a mixture of plasmids, encoding 1) for firefly luciferase, under the control of Thymidine Kinase minimal promoter and 2) firefly luciferase under the control of EF1 promoter ± the TRIB1 3’UTR. Twenty-four hours post-transfection, media was removed and cells were washed with PBS 1X twice and then lysed using 35 ml of 1X Passive Lysis Buffer (Promega); 5 ml of lysate was transferred onto a Nunc 384-well polystyrene white microplate. The substrates of Firefly luciferase (LAR II, Promega) and Renilla luciferase (Stop & Glo, Promega) were sequentially added to the cell lysates (1:1 ratio). From the same sample, luminescence was measured first at 560 nm for firefly luciferase and at 480 nm for Renilla luciferase using a microplate reader (Thermo Fisher Scientific). Each condition was plated in triplicate. All the readings were first normalized to the readings generated by non-transfected cells to subtract the luminescence background and then Renilla/Firefly ratio was calculated.

### Cholesterol Efflux Assay

Cholesterol efflux assay was performed on MDMs transfected with miR-101-3p mimic and inhibitor, 24 h post-transfection. Cells were treated with 2.5 uM of TopFluor Cholesterol (23-(dipyrrometheneboron difluoride)-24-norcholesterol (Avanti Polar Lipids) for 24 h in RPMI 0.2% BSA fatty-acid free (Sigma-Aldrich). Media was changed to only RPMI 0.2% BSA fatty-acid free for 18 h (equilibration step). After that, 50 µg/ml of High-Density Lipoprotein (HDL) (Biorad) was added as cholesterol acceptor for 4 h. The media (supernatant) was collected and cells were lysed using a solution of 1% Cholic Acid in 100% Ethanol (Sigma-Aldrich). Both cell lysates and supernatants were then added to a Nunc 96-well plate (black) in triplicates and fluorescence was read using a microplate reader (Thermo Fisher Scientific) (excitation 490 nm, emission 520 nm). Data were analyzed as described by Low et al. (28).

### Statistical Analysis

All experiments were performed at least three times. Graphs and statistical analysis were generated in GraphPad Prism 8.00 (GraphPad). Statistical methods are specified in each figure legend. P‐values <0.05 were considered to be statistically significant. Where appropriate, post-hoc power calculations have confirmed the likely lack of type II error. Group sizes are described in figure legends and, in line with conventions, for primary cells, each N represents an independent, healthy donor.

## Results

### Manipulation of TRIB1 Expression Alters Liver and Adipose Tissue Macrophage Phenotype *In Vivo* and Inflammatory Markers in Monocyte-Derived Macrophages *In Vitro*

**Figure 1:**
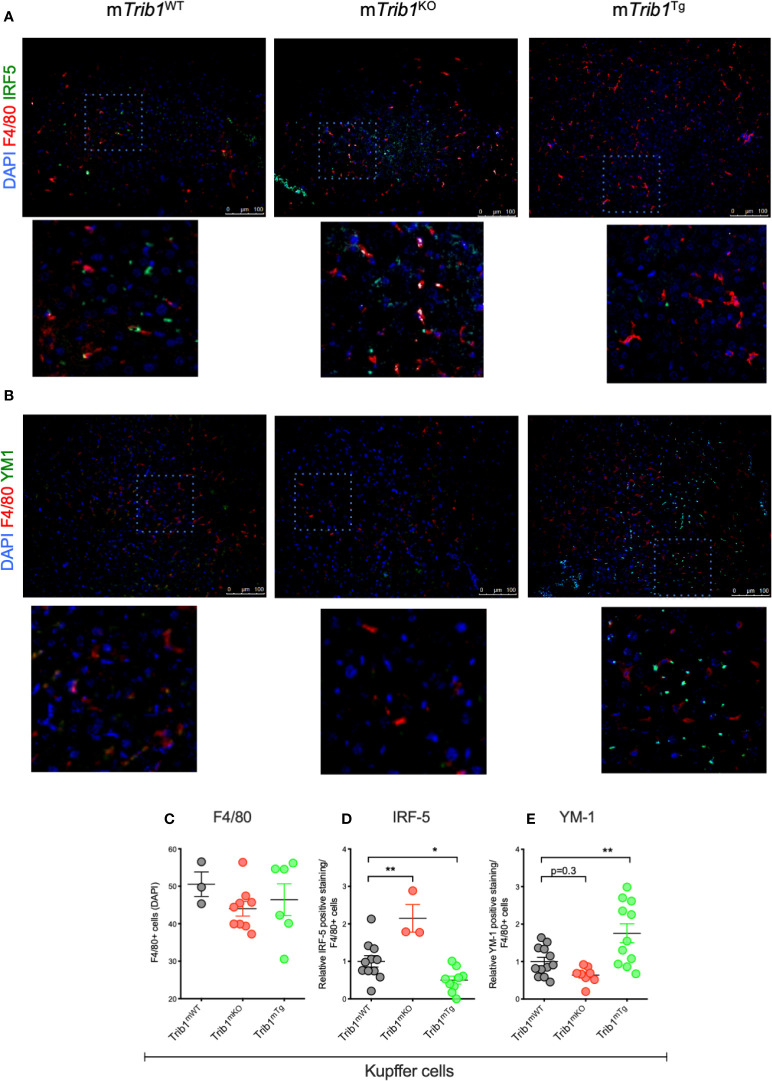
Genetic manipulation of myeloid Trib1 alters Kupffer cells phenotype *in vivo*. **(A, B)** Representative images of IRF5 and YM1 immunostaining of liver tissue sections from *Trib1^m^*^WT^, *Trib1^m^*^KO^ and *Trib1^m^*^Tg^ mice. Scale: 100 µm. Enlarged image of marked area is also shown. **(C)** Quantification of F4/80 positive cells relative to DAPI in liver tissue sections from *Trib1^m^*^WT^, *Trib1^m^*^KO^, and *Trib1^m^*^Tg^ mice (n = 3–9, ordinary one-way ANOVA with Sidak’s post-test, no statistical significance p > 0.05). **(D)** Quantification of IRF5 positive cells relative to F4/80 in liver tissue sections from *Trib1^m^*^WT^, *Trib1^m^*^KO^, and *Trib1^m^*^Tg^ mice (n = 3–11, ordinary one-way ANOVA with Sidak’s post-test, **p < 0.01, *p < 0.05). **(E)** Quantification of YM1 positive cells relative to F4/80 in liver tissue sections from *Trib1^m^*^WT^, *Trib1^m^*^KO^ and *Trib1^m^*^Tg^ mice (n = 8–12, ordinary one-way ANOVA with Sidak’s post-test, **p < 0.01, no statistical significance p > 0.05). Data are presented as mean ± SEM. Each data point (n) represents an individual mouse. At least three independent fields of view were examined per mouse. Samples were obtained and analyzed from three independent breeding cohorts, in total, each including WT littermate controls.

**Figure 2:**
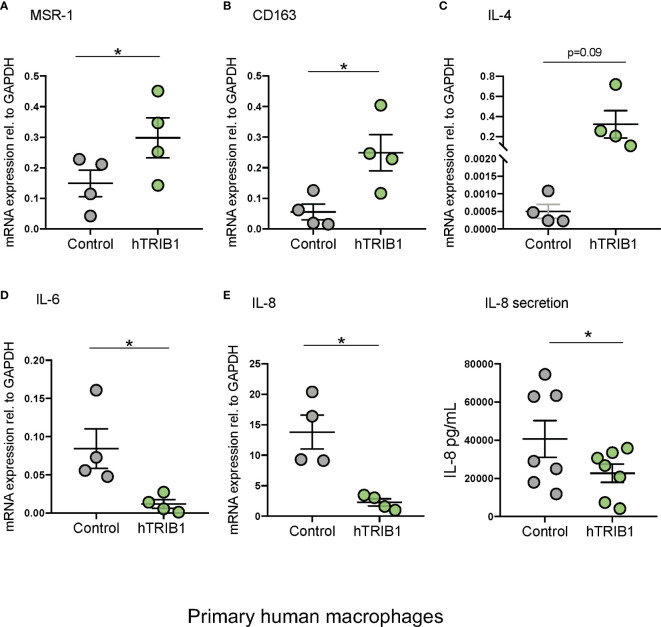
Genetic manipulation of TRIB1 alters macrophage phenotype *in vitro*. Relative mRNA expression of **(A)** MSR1, **(B)** CD163, **(C)** IL-4, **(D)** IL-6, and **(E)** IL-8 normalized to the housekeeping GAPDH and F: IL-8 (pg/ml) ELISA measured in MDMs transiently transfected with a TRIB1 overexpression plasmid and a control plasmid. Data are presented as mean ± SEM (scatter dot plot) (n = 4–7 biological replicates, paired t-test, *p < 0.05, no statistical significance p > 0.05). Transfection was carried out for 24 h, employing 2 µg of plasmid per well (6 well plate format). Each biological replicate represents the average of 3 technical replicates. ELISA was performed on supernatants collected 24 h post-transfection. RT-qPCR data were analyzed using the 2(−Δct) method.

We first determined the effect of myeloid-specific *Trib1* overexpression and KO on tissue-resident macrophage numbers and phenotype, employing wild-type (WT) and myeloid-specific *Trib1* knock-out and *Trib1* transgenic (Tg) mice. The generation and characterization of these strains has been reported previously (9). First, we wanted to ascertain the consequences of altered myeloid *Trib1* levels in metabolic tissues as this gene has been identified as a risk locus for hyperlipidaemia in a number of GWAS analyses (29–31) but its molecular mechanism impacting on lipid homeostasis is still incompletely understood. Dual immunofluorescence staining of Kupffer cells (liver resident macrophages) (**Figures 1A, B**) and adipose-tissue macrophages (ATMs) (**Supplementary Figure 2**), revealed that while altered *Trib1* expression has no effect on the number of F4/80 positive macrophages in these tissues (**Figure 1C** and **Supplementary Figure 2B**), it significantly altered their phenotype (**Figures 1D, E** and **Supplementary Figure 2C**). Specifically, Kupffer cells from *Trib1^mKO^* mice showed a significantly elevated expression of the pro-inflammatory transcription factor IRF-5 (n = 3), compared to Trib1^mWT^ (p = 0.02), while macrophages from *Trib1^mTg^* mice were characterized by a ~50% reduction of IRF-5 positive macrophages, compared to *Trib1^mWT^* (p = 0.04) (**Figure 1D**). *Trib1^mKO^* also exhibited a reduced expression of the anti-inflammatory marker YM-1 (~40% decrease on average), but this was not statistically significant (p = 0.3). Conversely, macrophages from *Trib1^mTg^* showed a ~75% increase in the expression of YM-1, compared to Trib1^mWT^ (p = 0.007) (**Figure 1E**). Similarly, ATM from *Trib1^mKO^* mice exhibited a ~50% reduction of YM-1 expression, compared to *Trib1^mWT^* (p = 0.01). However, *Trib1^mTg^* only had a modest, non-significant effect (~16% increase) on YM-1 expression in ATM (p = 0.4) (**Supplementary Figure 2C**), suggesting that *Trib1*-dependent changes in macrophage phenotypes may be modulated by the local tissue environment. Next, we evaluated the impact of *TRIB1* overexpression on the phenotype of human macrophages, employing transient transfection of monocyte-derived macrophages (MDMs) with a plasmid construct to overexpress the human TRIB1 protein. In response to *TRIB1* overexpression (shown in **Supplementary Figure 2**). We observed a significant increase in the mRNA levels of MSR1 (p < 0.05) and CD163 (p < 0.05), genes associated with alternatively activated macrophages (**Figures 2A, B**). Similarly, we observed an increase in IL-4, on average, but the change was not statistically significant (p = 0.09) (**Figure 2C**). Reduced levels of the pro-inflammatory cytokines IL-6 and IL-8 were also observed (**Figures 2D, E**). In addition, we measured the levels of IL-8 protein secreted in the media by MDMs and found that there was a significant reduction in IL-8 production by *TRIB1* overexpressing cells, compared to the control (**Figure 2E**). These data are consistent with previous reports (8, 32), indicating that myeloid *TRIB1* expression regulates the polarization of macrophages, favoring an anti-inflammatory, alternatively activated macrophage phenotype both *in vivo* as shown in *Trib1*-deficient mice (8) and in *in vitro*, as demonstrated in bone marrow–derived macrophages from Trib1 deficient mice (32).

### *TRIB1* Is Post-Transcriptionally Regulated by miRNAs

**Figure 3:**
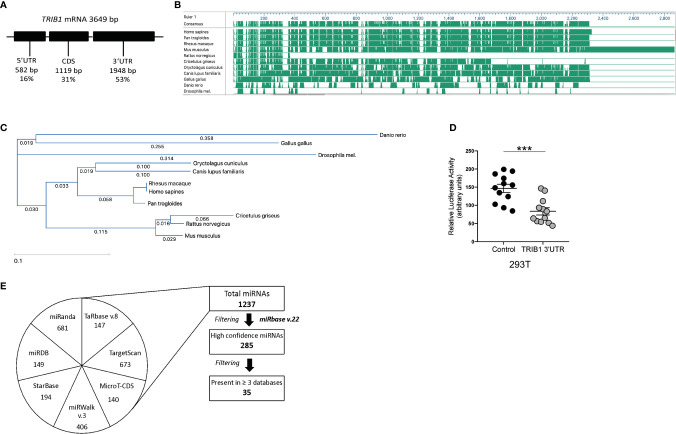
TRIB1 3’ UTR encodes for evolutionarily conserved miRNA binding sites. **(A)** Schematic representation of TRIB1 mRNA, showing the length of the untranslated regions and the protein coding sequence. **(B)** Schematic overview of TRIB1 3’UTR multi-species alignment, generated with the software DNASTAR Lasergene (v16). **(C)** TRIB1 3’UTR alignment tree, generated with the software DNASTAR Lasergene (v16) using the ClustalW algorithm. **(D)** Relative luciferase activity measured in HEK293T cell line after transfection of renilla luciferase reporter (control, 95 ng/well) and TRIB1 3’UTR renilla luciferase reporter (TRIB1 3’UTR, 95 ng/well) along with a firefly luciferase reporter plasmid (5 ng/well), used for data normalization (n = 12, biological replicates, unpaired t-test, ***p < 0.001). **(E)** Overview of miRNAs-target prediction analysis: seven different tools (miRanda, TargetScan, StarBase, miRDB, miRwalk, TarBase, and MicroT-CDS) were used to predict miRNAs targeting the 3’UTR of TRIB1; the list of distinct miRNAs was filtered selecting only “high-confidence” miRNAs (according to miRbase v.22) and miRNAs predicted at least by three different tools.

**Figure 4:**
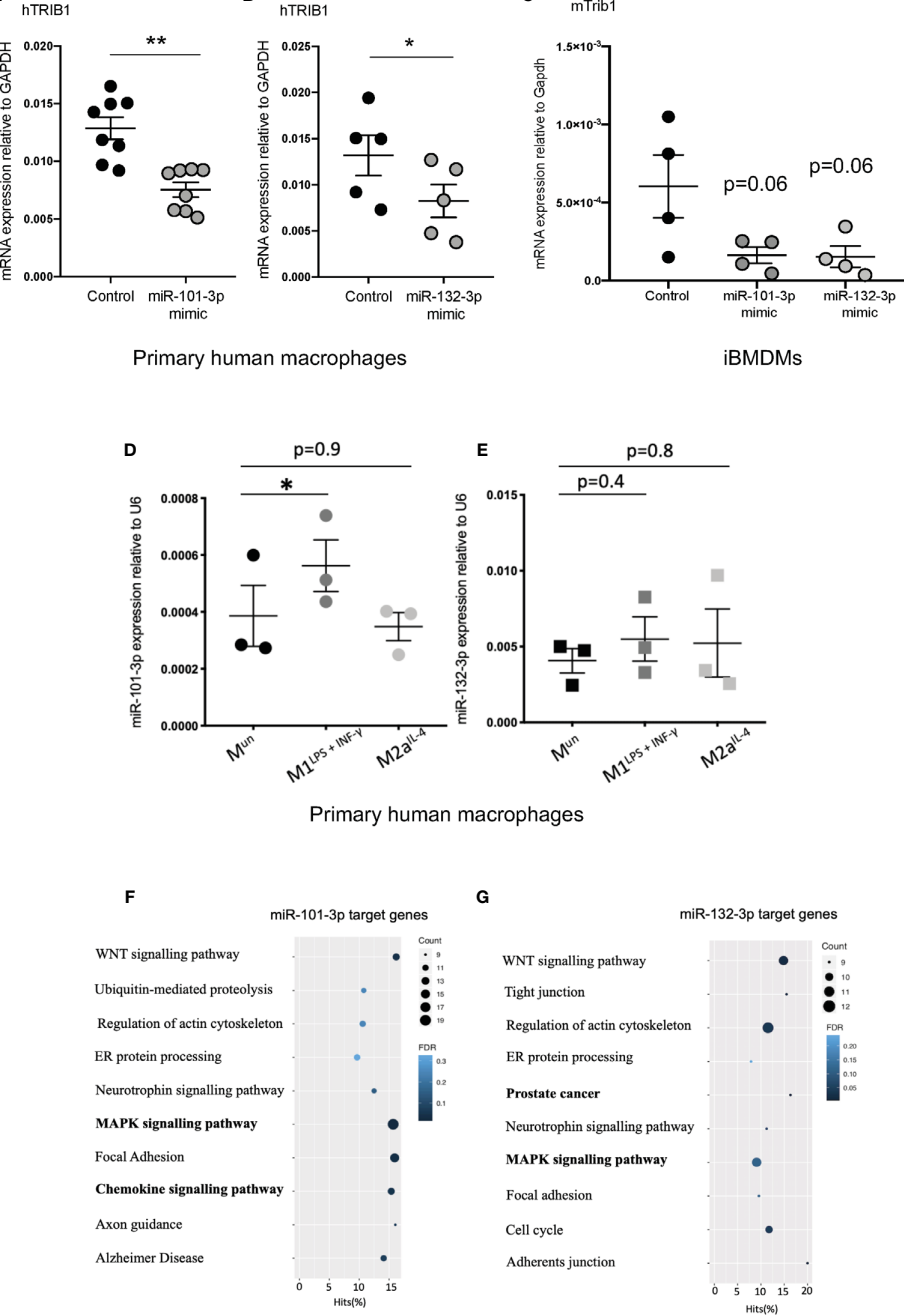
TRIB1 is post-transcriptionally regulated by miRNAs. **(A, B)** Relative mRNA expression of TRIB1 normalized to the housekeeping GAPDH in MDMs transiently transfected with miR-101-3p and miR-132-3p mimics (n = 5–8, biological replicates, paired t-test, **p < 0.01, *p < 0.05). **(C)** Relative mRNA expression of murine Trib1 normalized to the housekeeping Gapdh in iBMDMs transiently transfected with miR-101-3p and miR-132-3p mimics (n = 4, biological replicates, unpaired t-test, no statistical significance p > 0.05). **(D, E)** Relative expression of miR-101-3p and miR-132-3p normalized to the housekeeping U6 in unpolarized and M1- and M2- polarized MDMs (n = 3, biological replicates, repeated measure one way ANOVA with Dunnett’s post-test, *p < 0.05, no statistical significance p > 0.05). **(F, G)** KEGG enrichment pathway analysis of miR-101-3p and miR-132-3p target genes, predicted by using TargetScan and selecting macrophage-specific genes (from RNA-seq deposited at DOI: 10.17632/j2hmt7k9fh.1). Data are presented as mean ± SEM (scatter dot plot, where each dot represents an individual donor of MDMs or different passage of iBMDMs). Transient transfection of both DNAs and RNAs was carried out for 24 h. Mimics/control were used at 50 nM. RT-qPCR data were analyzed using the 2(−ΔCt) method. KEGG analysis was performed with the GOseq R package.

As *TRIB1* mRNA has been reported to be highly unstable (12), we investigated its post-transcriptional regulation by miRNAs. The mRNA of *TRIB1* encodes for a long and conserved 3’UTR region, representing more than half of the entire sequence (**Figure 3A**). Additionally, the 3’UTR sequence is well conserved among different animal species, as shown in **Figures 3B, C**. As the majority of miRNAs have functional target sites in this region, we evaluated the impact of the 3’UTR of *TRIB1* on mRNA stability using a luciferase reporter assay. Cloned downstream of the renilla luciferase gene, the 3’UTR of *TRIB1* led to a robust and significant decrease (> 40%) of the luciferase activity, compared to the control lacking the UTR (p < 0.0001) (**Figure 3D**), suggesting that this region contributes to the post-transcriptional regulation of *TRIB1*. This is in line with our previous findings where we showed that overexpression of *TRIB1* 3’UTR protects the endogenous mRNA, probably *via* titrating out factors (miRNAs or RNA binding proteins) that regulate the stability of the mRNA (2). To characterize the extent by which the effect of *TRIB1* 3’UTR may be mediated by interaction with miRNAs, we carried out a comprehensive *in silico* analysis using multiple miRNA-target prediction tools, including StarBase which is supported by CLIP-seq experimental data (http://starbase.sysu.edu.cn/) (**Figure 3E**). This analysis predicted that the 3’UTR of *TRIB1* can potentially interact with a total of 1,237 distinct miRNAs. Amongst these, 285 high confidence miRNAs were identified in miRbase v.22 (ftp://mirbase.org/pub/mirbase/CURRENT/). Annotation of high confidence miRNAs was based on RNA deep sequencing data and pattern of mapped reads, as described in details by Kozomara and colleagues (25); 35 high confidence miRNAs were predicted to bind to the *TRIB1* 3’UTR by at least 3 prediction tools (**Supplementary Table 6**). Next, we selected miR-101-3p and miR-132-3p for experimental validation: they were listed as high-confidence miRNAs and were predicted by the majority of the tools that we used, also showing evolutionarily conserved binding-sites in the 3’UTR of *TRIB1* mRNA (**Figures 3B, C** and **Supplementary Figure 4**). Additionally, they have been previously implicated in the literature to regulate inflammation and macrophage function (**Supplementary Table 7**). To determine the impact of these miRNAs on *TRIB1* expression, we transiently transfected miRNA mimics and a negative control into human MDMs (**Supplementary Figure 5**) and immortalized murine bone marrow–derived macrophages (iBMDMs). The endogenous mRNA expression of *TRIB1* was significantly reduced by miR-101-3p (p = 0.001) and miR-132-3p (p = 0.02), compared to the control (**Figures 4A, B**). Murine *Trib1* mRNA was also downregulated, but the changes were not statistically significant (**Figure 4C**). Next, we investigated the endogenous expression of these miRNAs in human unpolarized (Mun) and polarized (M1 and M2a) macrophages by using RT-qPCR. We found that miR-101-3p was upregulated in M1 but not in M2 macrophages, compared to unpolarized cells (p = 0.04) (**Figure 4D**). The expression of miR-132-3p did not change in either M1 or M2 macrophages (**Figure 4E**). To investigate the biological relevance of candidate miRNAs, we performed a KEGG enrichment pathway analysis on the subset of target genes for each miRNA, focussing on those that are expressed in human macrophages. To this aim, we used an RNA seq dataset performed on human polarized macrophages, previously generated in our group (DOI: 10.17632/j2hmt7k9fh.1). Interestingly, we found shared enriched terms between miR-101-3p and miR-132-3p target genes, including MAPK signaling pathway (**Figures 4F, G**), which is well established to control macrophage inflammatory responses (33) and is also regulated by TRIB1 (34–36). Additionally, we found in our KEGG analysis (**Figure 4G**) that several miR-132-3p target genes expressed in macrophages, have been suggested to be involved in prostate cancer, a disease in which *TRIB1* has been implicated (10, 11, 37). Additionally, it is well established that miR-132-3p acts as an onco-suppressor miR in prostate cancer, as several publications have reported (38, 39).

### miR-101-3p Has a Functional Binding Site in the 3’UTR of *TRIB1*

**Figure 5:**
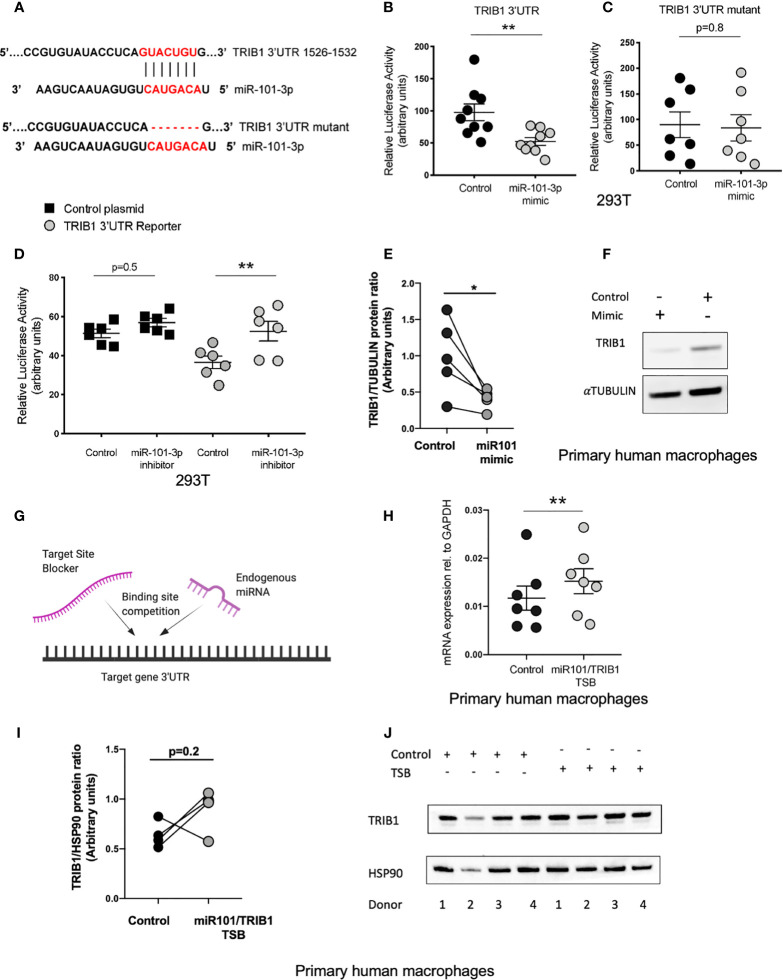
miR-101-3p has a functional binding site in the 3’UTR of TRIB1. **(A)** Schematic alignment of miR-101-3p with the 3’UTR of TRIB1 and its “binding site” mutant. **(B)** Relative luciferase activity measured in HEK293T cell line after co-transfection of the TRIB1 3’UTR renilla reporter (95 ng/well) with miR-101-3p mimic and a negative control (+firefly luciferase reporter, 5 ng/well), (n = 9, biological replicates, unpaired t-test, **p < 0.01). **(C)** Relative luciferase activity measured in HEK293T cell line after co-transfection of the TRIB1 3’UTR renilla reporter “mutant” (95 ng/well) with miR-101-3p mimic and a negative control (+ firefly luciferase reporter, 5 ng/well), (n = 7, biological replicates, unpaired t-test, no statistical significance p > 0.05). **(D)** Relative luciferase activity measured in HEK293T cell line after co-transfection of the renilla luciferase reporter (control, 95 ng/well) and TRIB1 3’UTR renilla reporter (TRIB1 3’UTR, 95 ng/well) with miR-101-3p inhibitor and a negative control (+ firefly luciferase reporter, 5 ng/well), (n = 6, biological replicates, ordinary one-way ANOVA with Sidak’s post-test, **p < 0.01, no statistical significance p > 0.05). **(E, F)** Trib1 protein levels relative to the housekeeping α-tubulin in MDMs transiently transfected with miR-101-3p mimic/negative control and representative immunoblot (n = 5, biological replicates, paired t-test, *p < 0.05). **(G)** Schematic representation of TSB/endogenous miRNA competition for the target site. **(H)** Relative TRIB1 mRNA expression normalized to the housekeeping GAPDH in MDMs transiently transfected with miR-101/TRIB1 TSB and a negative control (n = 7, biological replicates, paired t-test, **p < 0.01). **(I, J)** Trib1 protein levels relative to the housekeeping Hsp90 in MDMs transiently transfected with miR-101/TRIB1 TSB/negative control and representative immunoblot (n = 4, biological replicates, paired t-test, no statistical significance p > 0.05). Data are presented as mean ± SEM (scatter dot plot where each dot represents an individual donor of MDMs or different passages of HEK293T cells). Each biological replicate represents the average of 3 technical replicates. Transient transfection of both DNAs and RNAs was carried out for 24 h. Mimic/control, inhibitor/control and TSB/control were used at 50, 25, and 50 nM, respectively. RT-qPCR data were analyzed using the 2(−Δct) method.

miR-101-3p, a miRNA known to regulate inflammatory responses in monocyte/macrophages (40, 41), was predicted to bind to the 3’UTR of *TRIB1* between positions 1526-1532 (7mer-m8 site) and 1424-1430 (7mer-A1 site) (**Supplementary Table 7**). However, we focussed on the 7mer-m8 site, as it was predicted by all the tools used and was the most conserved (**Supplementary Table 7**); this is an established factor of likely biological relevance (19). Furthermore, the interaction is characterized by one of the most effective canonical seed region types, an exact match to position 2–8 of the mature miRNA (**Figure 5A**), which strongly correlates with targeting efficiency (19, 42). To substantiate the direct interaction between miR-101-3p and *TRIB1* 3’UTR, we co-transfected the *TRIB1* 3’UTR reporter plasmid and 50nM of miR-101-3p mimic or negative control in HEK293T cells and carried out a luciferase reporter assay. Overexpressing miR-101-3p led to a significant reduction of the *TRIB1* 3’UTR reporter activity compared to control (~45% reduction, p = 0.006) (**Figure 5B**). In contrast, miR-101-3p overexpression had no effect on reporter expression with a *TRIB1* 3’UTR mutant lacking the 7 nucleotides complementary to the miR-101-3p seed region (p = 0.8) (**Figures 5A, C**), confirming that the predicted miR-101-3p binding site between positions 1526-1532 is in fact targeted by miR-101-3p. Conversely, a miRNA inhibitor, which is designed to antagonize the activity of the endogenous miR-101-3p, led to a significant increase of the luciferase activity (~16% increase, p = 0.005) and had no effect when co-transfected together with a control plasmid, without the 3’UTR of *TRIB1* (p = 0.4) (**Figure 5D**). Next, we evaluated the effect of miR-101-3p mimic on endogenous TRIB1 protein levels in transfected human MDMs, as we previously showed that the mRNA of both human and murine *TRIB1* was negatively downregulated (**Figures 5D, E**). Upon transfection of the mimic, TRIB1 protein was reduced by approximately 60%, compared to control (p = 0.03) (**Figures 5E, F**). Finally, we investigated whether the activity of miR-101-3p on endogenous *TRIB1* mRNA and protein levels in macrophages is due to their direct physical interaction. To this aim, we transiently transfected MDMs with a miR-101/*TRIB1* TSB. This is an antisense oligonucleotide designed to selectively compete with miR-101-3p for binding the *TRIB1* 3’UTR. It binds to the target with high affinity, preventing the endogenous miRNA from binding to the same region and without activating the RISC complex (**Figure 5G**). The TSB treatment caused a significant increase of *TRIB1* mRNA (~30%, p = 0.004) compared to control (**Figure 5H**), suggesting that a direct interaction between miR-101-3p and *TRIB1* occurs in human macrophages. However, we did not observe a significant increase in the Trib1 protein (p = 0.2). This was likely due to an individual donor, as shown in the line graph in **Figure 5I** and in the immunoblot (**Figure 5J**, donor 2). Nevertheless, our data indicate that miR-101-3p is a negative, direct regulator of *TRIB1* in macrophages.

### miR-101-3p Drives a Pro-Inflammatory Phenotype in Human Macrophages

**Figure 6:**
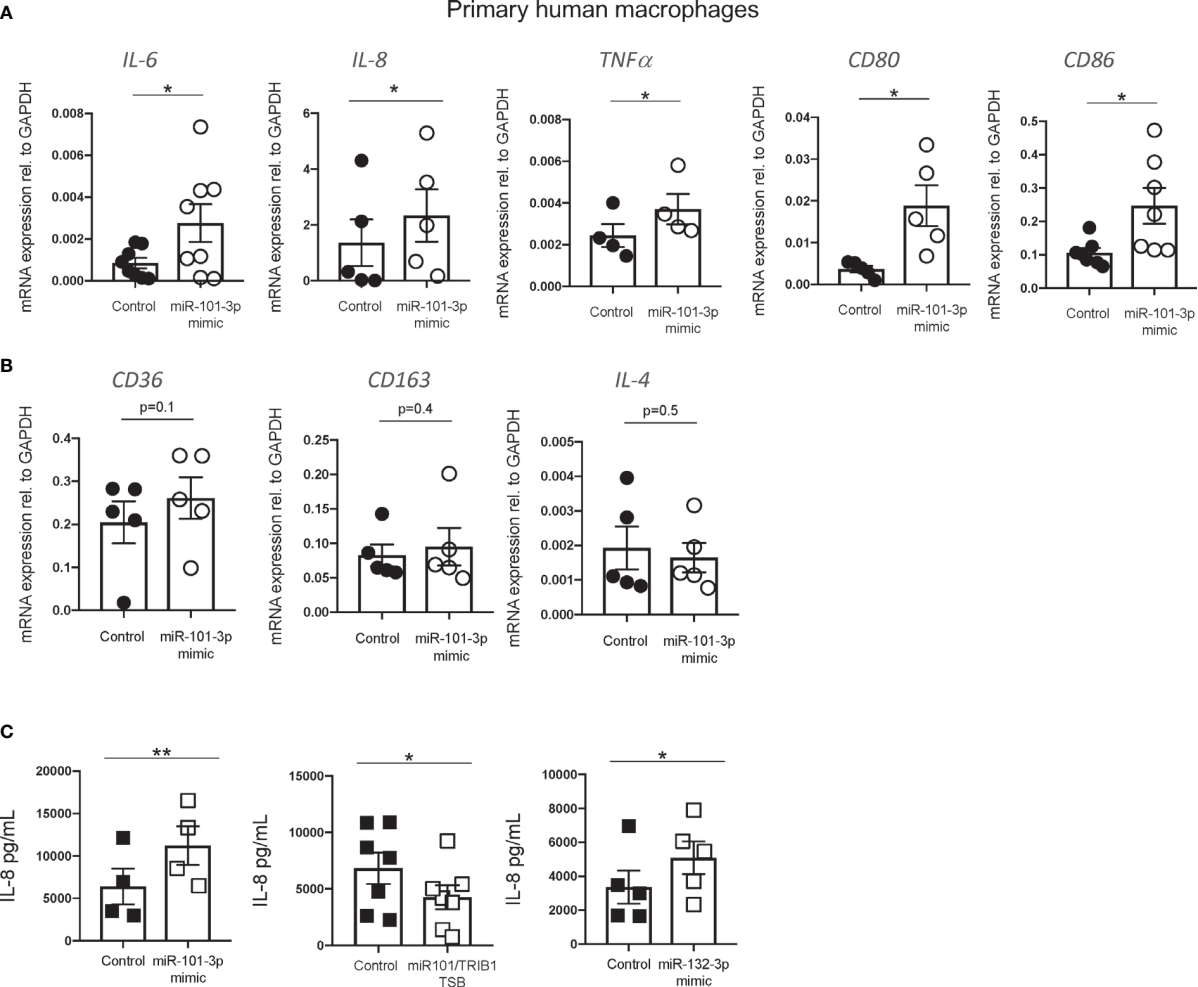
miR-101-3p drives a pro-inflammatory phenotype in human macrophages. **(A, B)** Relative mRNA expression of pro-inflammatory IL-6, IL-8, TNF-α c, CD80, and CD86 and anti-inflammatory macrophage markers CD36, CD163 and IL-4 normalized to the housekeeping GAPDH in MDMs transiently transfected with miR-101-3p mimic and a negative control. **(C)** Levels of IL-8 protein (pg/ml) measured from the supernatants of MDMs transiently transfected with miRNAs mimics and miR-101-3p/TRIB1 TSB. Data are presented as mean ± SEM (scatter dot plot where each dot represents an individual donor of MDMs). Each biological replicate represents the average of 3 technical replicates; (n = 4–8, biological replicates, paired t-test, **p < 0.01, *p < 0.05, no statistical significance p > 0.05). Transfection was carried out for 24 h, employing 50 nM of test and control RNAs. RT-qPCR data were analyzed using the 2(−Δct) method.

In macrophages, miR-101-3p was previously identified as a direct regulator of the dual specificity phosphatase 1 (*DUSP1*) (40) and ATP-binding cassette transporter A1 (*ABCA1*) (41) genes. This was associated with the activation of p38 and JNK and inhibition of cholesterol efflux under inflammatory and non-inflammatory conditions, respectively (40, 41). We confirmed the negative effect of miR-101-3p overexpression on *DUSP1* and *ABCA1* mRNA in unpolarized human MDMs (**Supplementary Figures 6A, B**) and its impact on cholesterol efflux to HDL (**Supplementary Figure 6C**). We next analyzed the expression of pro- and anti-inflammatory macrophage markers by RT-qPCR. We observed that miR-101-3p significantly increased the mRNA levels of IL-6, IL-8, TNF-α, CD80, and CD86 (**Figure 6A**), but had no effect on the anti-inflammatory markers CD36, CD163 and IL-4 (**Figure 6B**). We performed IL-8 ELISA on MDMs transfected with miR-101-3p mimic and miR-101/*TRIB1* TSB and found that the mimic enhanced IL-8 secretion (p = 0.007), while the TSB diminished it (p = 0.01) (**Figure 6C**). Additionally, we tested the impact of miR-132-3p, which downregulated the mRNA of *TRIB1* in both human and murine macrophages (see **Figure 4B**). IL-8 secretion was significantly increased in response to miR-132-3p mimic (p = 0.01), suggesting that *TRIB1* is a regulator of IL-8, downstream of the miRNAs tested. Overall, we conclude that miR-101-3p and miR-132-3p drive an inflammatory phenotype in human macrophages, at least in part by targeting TRIB1.

### *TRIB1* Is a Target of miRNAs Downregulated in Prostate Cancer

**Figure 7:**
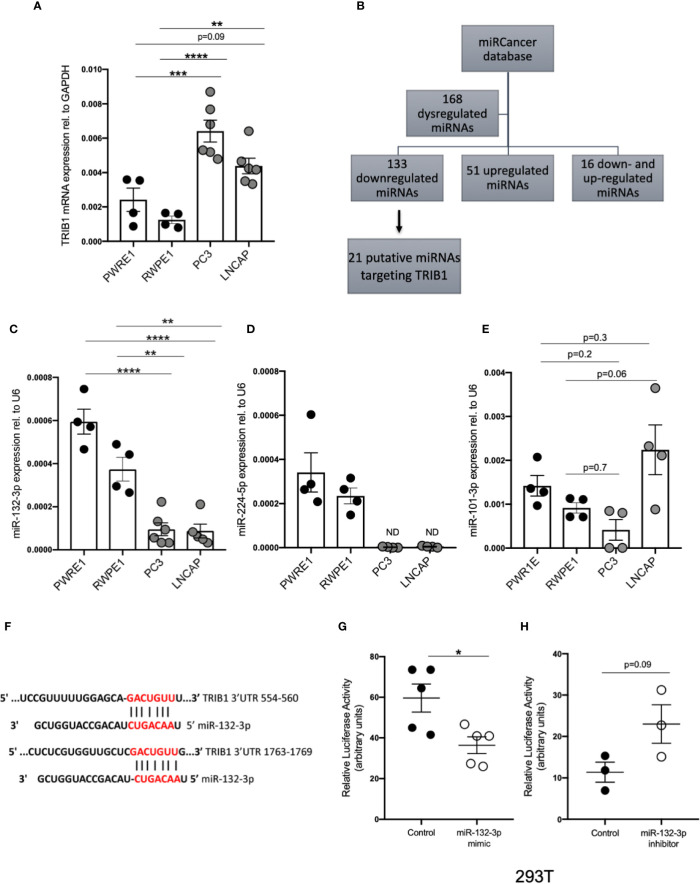
TRIB1 is a target of miRNAs downregulated in prostate cancer. **(A)** Relative mRNA expression of TRIB1 normalized to the housekeeping GAPDH in prostate cancer cell lines (n = 4–6, biological replicates, ordinary one-way ANOVA with Sidak’s post-test, ****p < 0.0001, ***p < 0.001, **p < 0.01, no statistical significance p > 0.05). **(B)** Flowchart for the detection of miRNAs downregulated/silenced in prostate cancer and potentially targeting the 3’UTR of TRIB1. **(C–E)** Relative expression of miR-132-3p, miR-224-5p and miR-101-3p normalized to the housekeeping U6 in a panel of prostate cancer and control cell lines (n = 4–6, biological replicates, ordinary one-way ANOVA with Sidak’s post-test, ****p < 0.0001, **p < 0.01, ND not detected, ns p > 0.05). **(F)** Schematic representation of miR-132-3p binding sites in two different positions of TRIB1 3’UTR. **(G, H)** Relative luciferase activity measured in HEK293T cell line after co-transfection of co-transfection of the TRIB1 3’UTR renilla reporter (95 ng/well) and miR-132-3p mimic/control (left) and miR-132-3p inhibitor/control (right) (+ firefly luciferase reporter, 5 ng/well) (n = 3–5, biological replicates, unpaired t-test, *p < 0.05, no statistical significance p > 0.05). Data are presented as mean ± SEM (scatter dot plot where each dot represents different passages of cell lines). Each biological replicate represents the average of 3 technical replicates. Transfection of plasmids and RNAs was carried out for 24 h. Mimic/control and inhibitor/control were used at 50 and 25 nM, respectively. RT-qPCR data were analyzed using the 2(−Δct) method.

**Figure 8:**
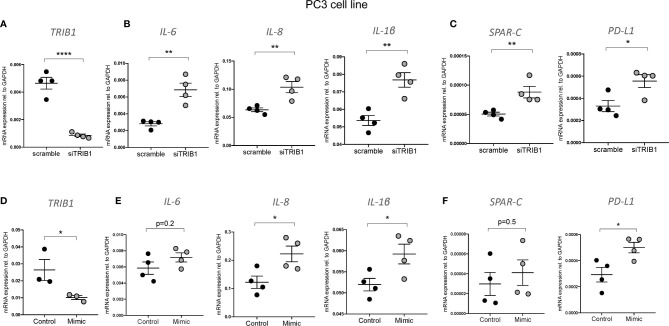
TRIB1 knock-down and miR-132-3p overexpression in PC3 cell lines. **(A**, **D)** Relative TRIB1 mRNA expression normalized to the housekeeping GAPDH in PC3 cell line transiently transfected with siTRIB1/Scramble and miR-132-3p/control (n = 3–4, biological replicates, unpaired t-test, ****p < 0.0001, *p < 0.05). **(B, C, E, F)** Relative mRNA expression of inflammatory markers and cancer-related genes normalized to the housekeeping GAPDH in in PC3 cell line transiently transfected with siTRIB1/Scramble and miR-132-3p/control (n = 4, biological replicates, unpaired t-test, **p < 0.01, *p < 0.05, no statistical significance p > 0.05). Data are presented as mean ± SEM (scatter dot plot where each dot represents a different passage of PC3 cells). Each biological replicate represents the average of three technical replicates. Transfection was carried out for 24 h. Mimic/control and siTRIB1/Scramble were used at 50 nM. RT-qPCR data were analyzed using the 2(−Δct) method.

It is widely reported that in prostate cancer the majority of dysregulated miRNAs are either silenced by epigenetic modifications or downregulated (43–45). At the same time, *TRIB1* has been reported to be upregulated in this cancer (10). To address the hypothesis that *TRIB1* upregulation occurs as a result of downregulation of endogenous miRNAs, we first assessed the endogenous *TRIB1* expression in *in vitro* models of prostate cancer. To this end we used PWR1E and RWPE1 as normal prostate epithelial cells and PC3 and LNCAP as cancer cell lines. The mRNA expression of *TRIB1* was significantly upregulated in PC3 and LNCAP cells, compared to RWPE1, non-cancer prostate epithelial cells (**Figure 7A**). Next, we used the miRCancer database to download the list of miRNAs reported to be dysregulated in prostate cancer and found that 21 downregulated miRNAs are also predicted to target the 3’UTR of *TRIB1*, according to TargetScan (**Figure 7B** and **Supplementary Table 7**). Among them, we selected miR-132-3p and miR-224-5p, since both of these miRNAs are established to be silenced in prostate cancer (38, 46). Additionally, as described above, miR-132-3p is a potential regulator of *TRIB1* in both human and murine macrophages and miR-224-5p has also been already implicated in the regulation of this pseudokinase in prostate cancer (47). Therefore, we assessed the expression of these miRNAs in PC3 and LNCAP cells. Considering our previous findings, we included miR-101-3p as it was also listed as miRNA downregulated in prostate cancer, according to the miRCancer database. miR-132-3p was significantly downregulated in both cell lines, compared to controls (**Figure 7C**), while miR-224-5p was not detectable in the cancer cells (**Figure 7D**). On the contrary, the endogenous expression of miR-101-3p in PC3 and LNCAP lines was not significantly different from the controls (**Figure 7E**). Therefore, we next focused on the activity of miR-132-3p. miR-132-3p has two predicted binding sites on the 3’UTR of *TRIB1* (**Figure 7F**). Despite not being highly conserved, the target sites are both characterized by a 7mer-m8 seed region (**Supplementary Table 7**). When tested in a luciferase assay, miR-132-3p mimic led to a significant reduction of the TRIB1 3’ UTR reporter activity, compared to control (p = 0.02) (**Figure 7G**). Transfection of the inhibitor led to an increase in the luciferase activity, on average, however this did not reach statistical significance (p = 0.09) (**Figure 7H**). We note that miRNA inhibitors are often less effective in rescuing miRNA-target expression, especially when the endogenous miRNAs they target are present at low levels. Next, we performed a transient transfection of PC3 cells with *TRIB1* siRNA/scramble and miR-132-3p mimic/control and analyzed the gene expression profile of pro-inflammatory cytokines and cancer-related genes and found similar signatures. The assessment of *TRIB1* mRNA downregulation induced by the siRNA and miR-132-3p is shown in **Figures 8A, D**, respectively. Either condition led to significant upregulation of pro-inflammatory cytokines (*IL-6, IL-8* and *IL-1β*) (**Figures 8B, E**), in line with our above presented data obtained in human macrophages. The immune checkpoint regulator gene, *PD-L1*, was also significantly increased in both conditions, while the extracellular matrix glycoprotein *SPAR-C* was enhanced only in PC3 cells treated with *TRIB1* siRNA (p = 0.01) (**Figures 8C, F**), hinting a role for TRIB1 in the regulation of cancer immune escape and invasion properties. Put together, these results suggest that the miR-132-3p/*TRIB1* axis may be a regulator of the immunological profile of prostate cancer.

## Discussion

Since their discovery 20 years ago (48, 49), Tribbles proteins have gained significant attention and have been implicated in many different pathologies, including cancer, cardiovascular disease, and metabolic disorders (5, 9, 10, 50–52). The role of *TRIB1* has been particularly studied in myeloid cells and adipose tissue, and linked to the regulation of anti-inflammatory macrophage polarization and inflammatory responses (7, 8, 32). Recently, it has emerged that *TRIB1* is often overexpressed in prostate cancer and is associated with cancer risk, but not with aggressiveness and survival (37). Although *TRIB1*-mediated signaling pathways are well characterized (1, 2, 34, 53), the upstream mechanisms underlying its regulation remain unknown. The observation that altered expression of *Trib1* in myeloid cells leads to significant changes in the phenotypes of liver and adipose macrophages provides further evidence for the importance of this pseudokinase in regulating metabolic homeostasis.

This prompted us to explore upstream factors, with focus on miRNAs, that may be important in regulating Trib1 expression levels in macrophages, as well as exploring whether similar regulatory mechanisms may control *TRIB1* levels in cellular models of PCa. Thus, we looked at the post-transcriptional regulation of *TRIB1*, with a focus on the importance of its 3’ UTR. This region of eukaryotic mRNAs has been extensively studied and several types of regulatory elements contributing to the control of mRNA (in)stability have been described ([a recent review, see (54)]. A study conducted in murine embryonic stem cells reported that *TRIB1* mRNA has a half-life shorter than 1 h and it was ranked among the top 50 most unstable genes (12). As the human *TRIB1* mRNA is characterized by a 2 kb long and conserved 3’UTR, we proposed that regulatory sequences within this may contribute to the control of *TRIB1* mRNA stability, including a role for miRNAs. In line with this hypothesis, we showed that the 3’UTR of *TRIB1* had a strong, negative effect on luciferase expression by using a reporter assay. Our *in-silico* analysis indicated that multiple “high-confidence” miRNAs are predicted to bind to the 3’UTR of *TRIB1*. So far, only miR-23a and miR-224-5p have been experimentally validated and shown to downregulate *TRIB1* expression in hepatocellular carcinoma and prostate cancer models, respectively (47, 55). Specifically, we demonstrated that miR-101-3p and miR-132-3p were able to negatively modulate both human and murine *TRIB1* mRNA expression in macrophages. To elucidate the biological impact of these miRNAs, we performed an enrichment pathway analysis using their predicted target genes, selecting only those expressed by human macrophages. Among the top 10 enriched terms for miR-101-3p target genes we observed MAPK and chemokine signaling pathways, both of which have previously been shown to be extensively regulated *via Trib1*. We demonstrated that the experimental overexpression of miR-101-3p in primary human macrophages led to a significant increase in the mRNA levels of pro-inflammatory markers, such as *IL-6*, *IL-8*, *TNF-α*, *CD80*, and *CD86*. It has been previously reported that *TRIB1* regulates the activity of the *IL-8* promoter in a gene reporter assay (2). More recently, it was shown that *TRIB1* negatively regulates *IL-8* secretion by inhibiting IKB-zeta in prostate cancer cells (11). To investigate the consequences of the putative interaction between endogenous miR-101-3p and *TRIB1* mRNA, we evaluated the impact of miR-101-3p on both *TRIB1* expression and IL-8 secretion by employing a TSB, a molecule designed to selectively prevent the endogenous miR from binding the mRNA of only *TRIB1*, without altering the miRNA expression itself. The TSB caused a significant increase in the steady-state mRNA of *TRIB1* and a reduction in IL-8 secretion. Similarly, we showed that miR-132-3p mimics downregulated *TRIB1* and enhanced secreted IL-8 protein levels. Therefore, our findings indicate that endogenous miRNAs are able to control *TRIB1* expression and, in turn, regulate *IL-8 via* targeting *TRIB1*. Of note, miR-132-3p was also shown to increase IL-8 levels in human adipocytes by targeting SirT1 and thus activating NF-κB (56), a key activator of inflammation and the latter also being a known TRIB1 target (7, 57). In light of our data, it is plausible that the miR-132-3p/*TRIB1* regulatory axis may play a part in this. However, we also explored the interaction between miR-132-3p and *TRIB1* in the context of prostate cancer. Our KEGG analysis of miR-132-3p predicted targets showed an enrichment of genes involved in prostate cancer. Although miR132 did not show the highest number of gene reads in PCa, we focused on this pathway because miR-132-3p is usually silenced by promoter hypermethylation in prostate cancer (38). A number of studies reported that restoring miR-132-3p expression in prostate cancer resulted in the suppression of cell proliferation, migration and invasion (38, 39, 58, 59). In addition to miR-132-3p, we identified 20 other miRNAs which are either silenced or downregulated in prostate cancer and also predicted to target *TRIB1*, with multiple binding sites, including miR-101-3p. Thus, we speculate that the downregulation of endogenous miRNAs could, at least in part, account for the elevated expression of *TRIB1* in prostate cancer, the causes of which have not been established yet (10, 37, 47). Here, we assessed the endogenous expression of miR-132-3p, miR-224-5p and miR-101-3p in a panel of prostate cancer cell lines and controls. We selected miR-132-3p and miR-101-3p as we validated their impact on *TRIB1* in human macrophages, and miR-224-5p as a positive control. In fact, miR-224-5p has previously been shown to be downregulated in prostate cancer and to target *TRIB1* and inhibit prostate cancer growth *in vitro* (47). We found that miR-132-3p and miR-224-5p were significantly downregulated in cancer cells compared to controls, while the expression of miR-101-3p did not show any significant changes among the different cell lines. As the interaction between TRIB1 and miR-224-5p has been functionally validated already (47), we focussed on miR-132-3p, showing that the downregulation of *TRIB1* by either a small interfering RNA or miR-132-3p mimic led to similar transcriptomic signatures in PC3 cells and confirmed the impact of the pseudokinase on *IL-6* and *IL-8* mRNA expression, even in non-myeloid cells. Of interest, *MSR1* expression has also been enhanced in the presence of overexpressed TRIB1. In addition to being a widely used marker of M2-like macrophages, this gene has also been linked to the development of prostate cancer (60, 61). The immune checkpoint regulator gene, *PD-L1* also increased in both conditions, suggesting a role for *TRIB1* in regulating the “immune escape” of prostate cancer. While our data presented in this paper provides evidence for TRIB1 altering macrophage phenotypes (pro- vs. anti-inflammatory) both in murine metabolic tissues and human monocyte-derived macrophages, further mechanistic studies are required to elucidate the full impact of these TRIB1-mediated changes on prostate cancer cells. However, our data supports the hypothesis that the role of TRIB1 in prostate cancer is, at least in part, linked to macrophage polarization and function, as a recent work has suggested (11). In fact, Liu and colleagues demonstrated that high levels of *TRIB1* in prostate cancer correlate with CD163^+^ macrophage infiltration. In addition, they showed that TRIB1 inhibits the secretion of cytokines from prostate cancer cells *via* inhibition of IKB-zeta. This, in turn, leads to the differentiation of anti-inflammatory M2-like macrophages in the surrounding microenvironment, which supports tumour growth (11). Of particular interest, a recent from Carracedo and his colleagues (62) demonstrates a cancer cell-intrinsic role for TRIB1 in prostate cancer by showing that *TRIB1* often overexpressed in PCa, and that overexpression of this gene in the prostate epithelium accelerates tumourigenesis in a mouse model. Thus, our data and the literature collectively suggest an important regulatory role for *TRIB1* both in macrophages and tumour cells, thus may provide a novel mechanism for the interplay between the tumour microenvironment and cancer cells.

In summary, in this report, we have demonstrated that *TRIB1* is subject to post-transcriptional regulation by miRNAs. By using a systematic miRNA-target prediction analysis, we identified a number of “high-confidence” miRNAs predicted to bind to the 3’UTR of *TRIB1* through a canonical seed region matching. We experimentally validated the interaction of the 3’UTR of *TRIB1* with miR-101-3p and miR-132-3p in human macrophages and prostate cancer, respectively, showing that they control *TRIB1* expression and, in turn, alter the mRNA and protein levels of *IL-8*. Of note, data we present on miRNA-132-3p action is lacking TSB blockade validation, similar to as shown for miRNA-101-3p, due to constrains of overall available primary macrophage cell numbers used in these experiments. However, we also recognize that the presented association between TRIB1-regulating miRNAs and the development of PCa is largely correlative in nature, thus future investigations will be required to further substantiate the biological relevance of these findings.

We also note that some of the data presented here is preliminary in nature and analysis of larger number of replicates have been severely hampered by the impact of COVID19 pandemic imposed restrictions. In addition to technical challenges of restricted working patterns, this disease is driving a severe monocyte/macrophage response is often asymptomatic or very mildly symptomatic in younger adults, thus controlling for potential effects of SARS-COV-2 in human monocyte-derived macrophages obtained from healthy volunteers that may interfere with our readouts is very challenging.

## Data Availability Statement

The original contributions presented in the study are included in the article/**Supplementary Material**. Further inquiries can be directed to the corresponding authors.

## Author Contributions

CN, OV, IS, HW, and EK-T conceived the experiments. CN, JJ, SD, SS, and ZS performed all the experiments and bioinformatics analysis. All authors contributed to the article and approved the submitted version.

## Funding

This work was funded by the European Marie Sklodowska Curie ITN Project TRAIN-TRIBBLES Research and Innovation Network (Grant No. 721532) and funds from the British Heart Foundation (PG/16/44/32146). We are very grateful to Veronika Kiss-Toth, Jonathan Kilby, Benjamin Durham, Markus Arians, Fiona Wright, and Yvonne Stephenson for their great technical support.

## Conflict of Interest

The authors declare that the research was conducted in the absence of any commercial or financial relationships that could be construed as a potential conflict of interest.

## Supplementary Material

Supplementary Figure 1 Immunofluorescence: isotype controls and ATM staining. FFPE adipose tissue from myeloid *Trib1* mice were stained with F4/80 (red) and the anti-inflammatory/M2 marker YM1 (green). Representative image of isotype controls and ATM staining is shown. Enlarged image of the boxed area is shown illustrating F4/80+ macrophage and dual positive (F4/80+ YM1+) macrophage.

Supplementary Figure 2 Genetic manipulation of myeloid Trib1 alters ATM phenotype *in vivo*. **(A)** Representative image of YM1 immunostaining of adipose tissue sections from *Trib1^m^*^WT^, *Trib1^m^*^KO^ and *Trib1^m^*^Tg^ mice. Scale: 100µm. Enlarged image of marked area is also shown. Each n represents the mean of at least 3 fields of view from an individual mouse. **(B)** Quantification of F4/80 positive cells in adipose tissue sections from *Trib1^m^*^WT^, *Trib1^m^*^KO^ and *Trib1^m^*^Tg^ mice. (n = 4–9 biological replicates, ordinary one-way ANOVA with Sidak’s post-test, no statistical significance p > 0.05). **(C)** Quantification of YM1 positive cells relative to F4/80 in adipose tissue sections from *Trib1^m^*^WT^, *Trib1^m^*^KO^ and *Trib1^m^*^Tg^ mice (n = 3–6, biological replicates, ordinary one-way ANOVA with Sidak’s post-test, *p < 0.05, no statistical significance p > 0.05). Each n represents an individual mouse; mice were obtained from two independent breeding cohorts and were stained per cohort, each including WT litter-mate controls. At least 3 independent fields of view were examined per mouse.

Supplementary Figure 3 Confirmation of TRIB1 overexpression in human macrophages. Relative TRIB1 mRNA expression normalized to the housekeeping GAPDH in MDMs transiently transfected with TRIB1 overexpression plasmid and a control plasmid (n = 4, biological replicates, paired t-test, no statistical significance p > 0.05). Data are presented as mean ± SEM (scatter dot plot where each dot represents an individual donor of MDMs). Biological replicate represents the average of 3 technical replicates. Transfection was carried out for 24 h, employing 2 µg of plasmid per well (6 well plate). RT-qPCR data were analyzed using the 2(−Δct) method.

Supplementary Figure 4 TRIB1 3’UTR mega alignment. Overview of TRIB1 3’UTR sequences alignment in different species, generated with the software DNASTAR Lasergene (v16); miR-101-3p and miR-132-3p binding sites are highlighted in red and black, respectively.

Supplementary Figure 5 Confirmation of miRNAs overexpression in human macrophages. Relative expression of miR-101-3p and miR-132-3p normalized to the housekeeping U6 in MDMs transiently transfected with miRNA mimics (n = 5–8, biological replicates, paired t-test, ***p < 0.001). Data are presented as mean ± SEM (scatter dot plot where each dot represents an individual donor of MDMs). Biological replicates represent the average of 3 technical replicates. Transfection was carried out for 24 h, employing 50nM of mimics and negative control. RT-qPCR data were analyzed using the 2(−Δct) method.

Supplementary Figure 6 Effect of miR-101-3p on previously validated targets: DUSP1 and ABCA1. **(A, B)** Relative mRNA expression of DUSP1 and ABCA1 normalized to the housekeeping GAPDH in MDMs transiently transfected with miR-101-3p mimic and a negative control (n = 4–7, biological replicates, paired t-test, **p < 0.01, *p < 0.05). **(C)** Percentage of total cholesterol efflux to HDL measured in MDMs transiently transfected with miR-101-3p mimic and inhibitor (n = 6, biological replicates, paired t-test, **p < 0.01). Data are presented as individual points **(A, B)** and as mean ± SEM (scatter dot plot) **(C)**. Each dot represents an individual donor of MDMs; biological replicates represent the average of 3 technical replicates. Transfection was carried out for 24 h, employing 50 nM of mimic/control and 25 nM of inhibitor/control. RT-qPCR data were analyzed using the 2(−Δct) method. As miRNA mimic and inhibitor were used at different concentrations and along with their controls (negative control mimic and negative control inhibitor) we used the paired t-test.

Supplementary Table 1 List of RNA constructs used for transient transfection.

Supplementary Table 2 List of primary and secondary antibodies used for western blot (WB) and immunofluorescence (IF) experiments.

Supplementary Table 3 List of RT-qPCR primers.

Supplementary Table 4 List of PCR primers used for site-directed mutagenesis.

Supplementary Table 5 List of “high-confidence” miRNAs predicted to target the 3’UTR of TRIB1 by 3 different prediction tools, along with their features taken from TargetScan. The list of “high-confidence” miRNAs can be accessed and downloaded using this link ftp://mirbase.org/pub/mirbase/CURRENT (miRbase v.22).

Supplementary Table 6 The table lists candidate miRNAs selected for experimental validation and their features, according to TargetScan; both were listed as “high-confidence” miRNAs, according to deep sequencing published data (miRbase v.22).

Supplementary Table 7 List of miRNAs downregulated/silenced in prostate cancer and predicted to target the 3’UTR of TRIB1. miRNA-target prediction analysis was performed by using TargetScan, miRanda and Starbase. The list of dysregulated miRNAs in prostate cancer was taken from miRCancer and it is accessible at this link: http://mircancer.ecu.edu/search.jsp?mirId=&logic=&condition=Or&cancerName=prostate+cancer&buttonSearch.
