# Supplementary figures and images for: Tribbles-1 Expression and Its Function to Control Inflammatory Cytokines, Including Interleukin-8 Levels are Regulated by miRNAs in Macrophages and Prostate Cancer Cells
Source: Front Immunol. 2020 Nov 27;11:574046. doi: 10.3389/fimmu.2020.574046 (PMC7728618; doi:10.3389/fimmu.2020.574046)

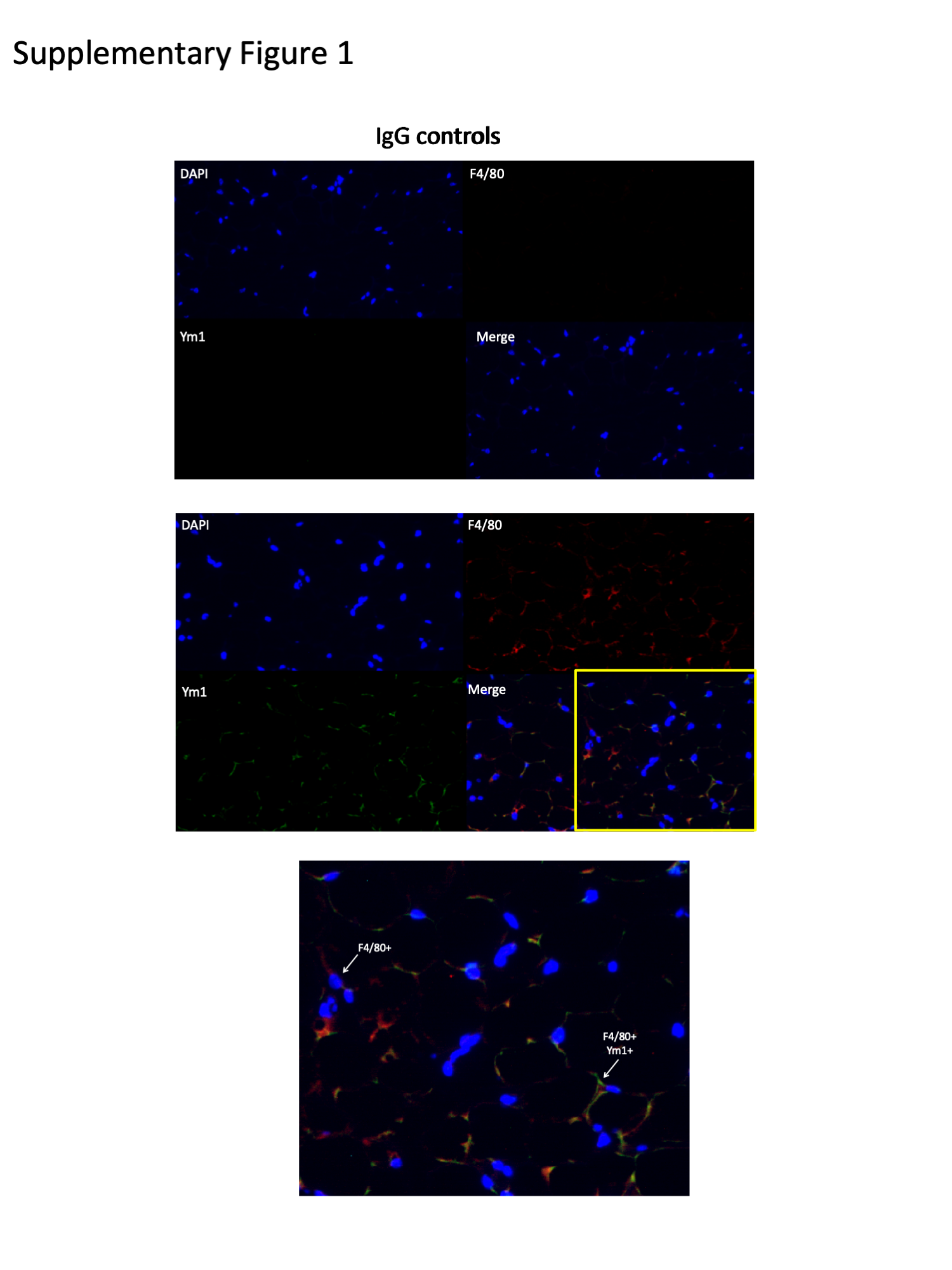

Supplement: Supplementary Figure 1 — Immunofluorescence: isotype controls and ATM staining. FFPE adipose tissue from myeloid Trib1 mice were stained with F4/80 (red) and the anti-inflammatory/M2 marker YM1 (green). Representative image of isotype controls and ATM staining is shown. Enlarged image of the boxed area is shown illustrating F4/80+ macrophage and dual positive (F4/80+ YM1+) macrophage. [file Image_1.tiff]

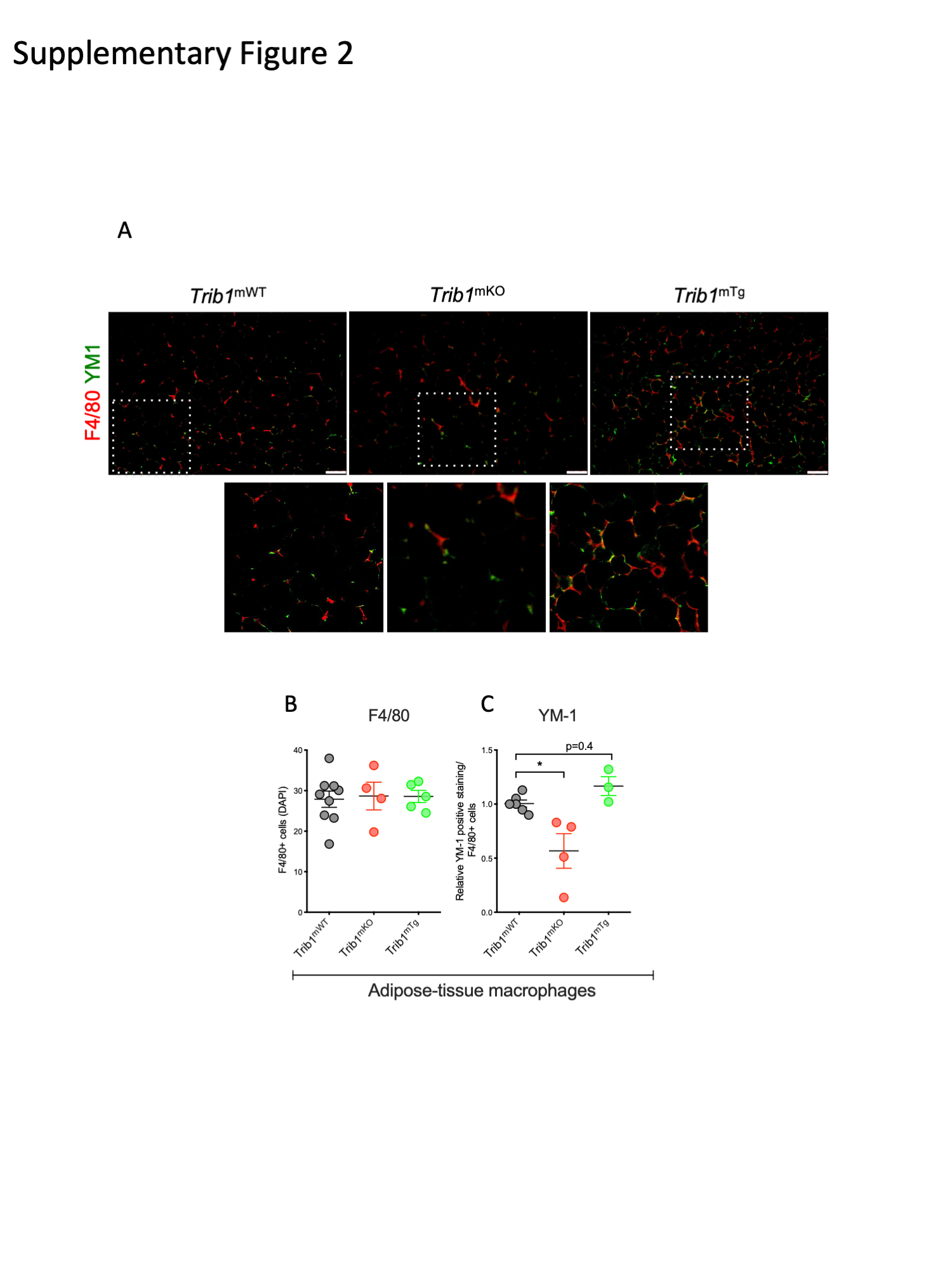

Supplement: Supplementary Figure 2 — Genetic manipulation of myeloid Trib1 alters ATM phenotype in vivo. (A) Representative image of YM1 immunostaining of adipose tissue sections from Trib1mWT, Trib1mKO and Trib1mTg mice. Scale: 100µm. Enlarged image of marked area is also shown. Each n represents the mean of at least 3 fields of view from an individual mouse. (B) Quantification of F4/80 positive cells in adipose tissue sections from Trib1mWT, Trib1mKO and Trib1mTg mice. (n = 4–9 biological replicates, ordinary one-way ANOVA with Sidak’s post-test, no statistical significance p > 0.05). (C) Quantification of YM1 positive cells relative to F4/80 in adipose tissue sections from Trib1mWT, Trib1mKO and Trib1mTg mice (n = 3–6, biological replicates, ordinary one-way ANOVA with Sidak’s post-test, *p < 0.05, no statistical significance p > 0.05). Each n represents an individual mouse; mice were obtained from two independent breeding cohorts and were stained per cohort, each including WT litter-mate controls. At least 3 independent fields of view were examined per mouse. [file Image_2.tiff]

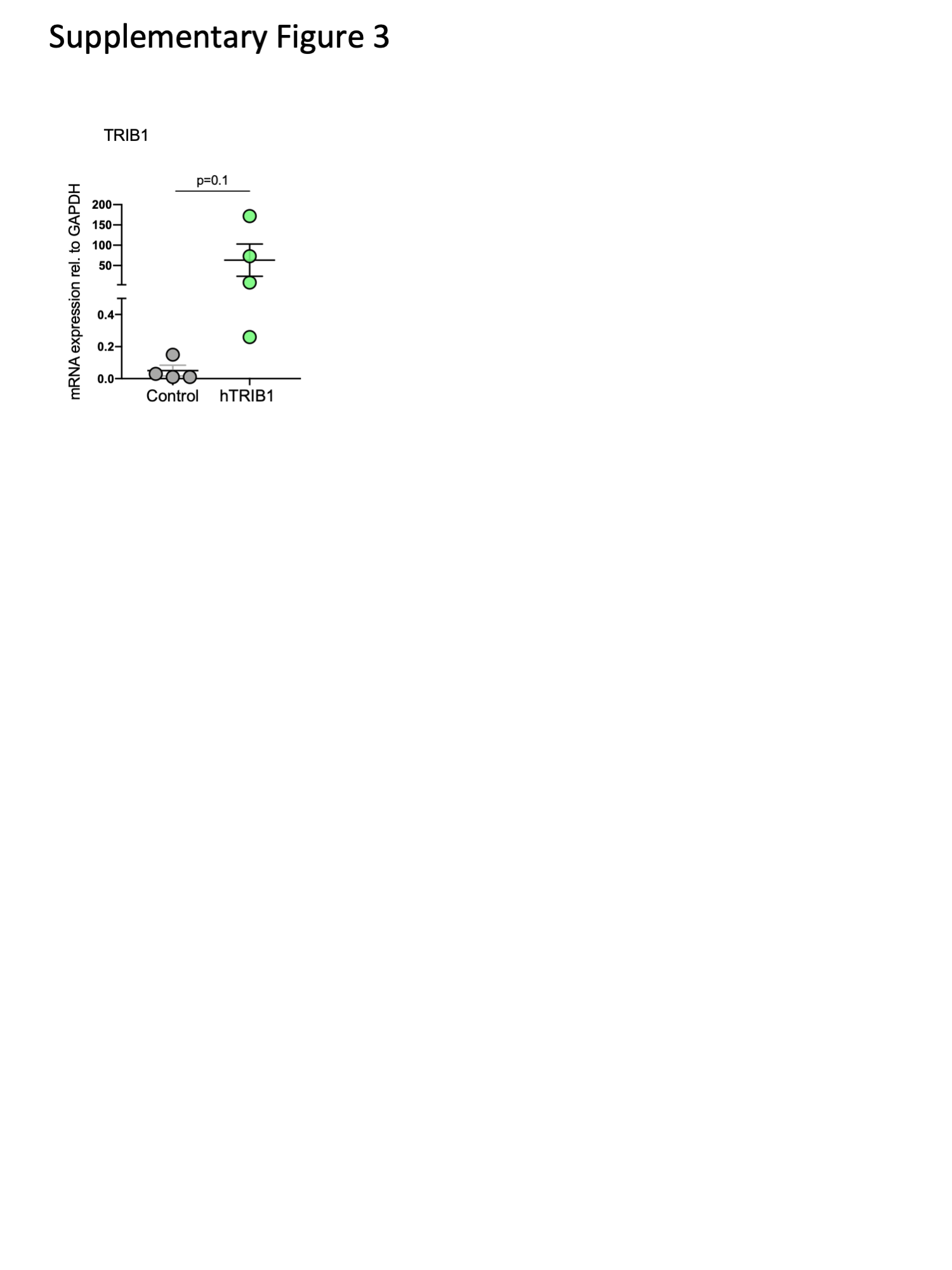

Supplement: Supplementary Figure 3 — Confirmation of TRIB1 overexpression in human macrophages. Relative TRIB1 mRNA expression normalized to the housekeeping GAPDH in MDMs transiently transfected with TRIB1 overexpression plasmid and a control plasmid (n = 4, biological replicates, paired t-test, no statistical significance p > 0.05). Data are presented as mean ± SEM (scatter dot plot where each dot represents an individual donor of MDMs). Biological replicate represents the average of 3 technical replicates. Transfection was carried out for 24 h, employing 2 µg of plasmid per well (6 well plate). RT-qPCR data were analyzed using the 2(−Δct) method. [file Image_3.tiff]

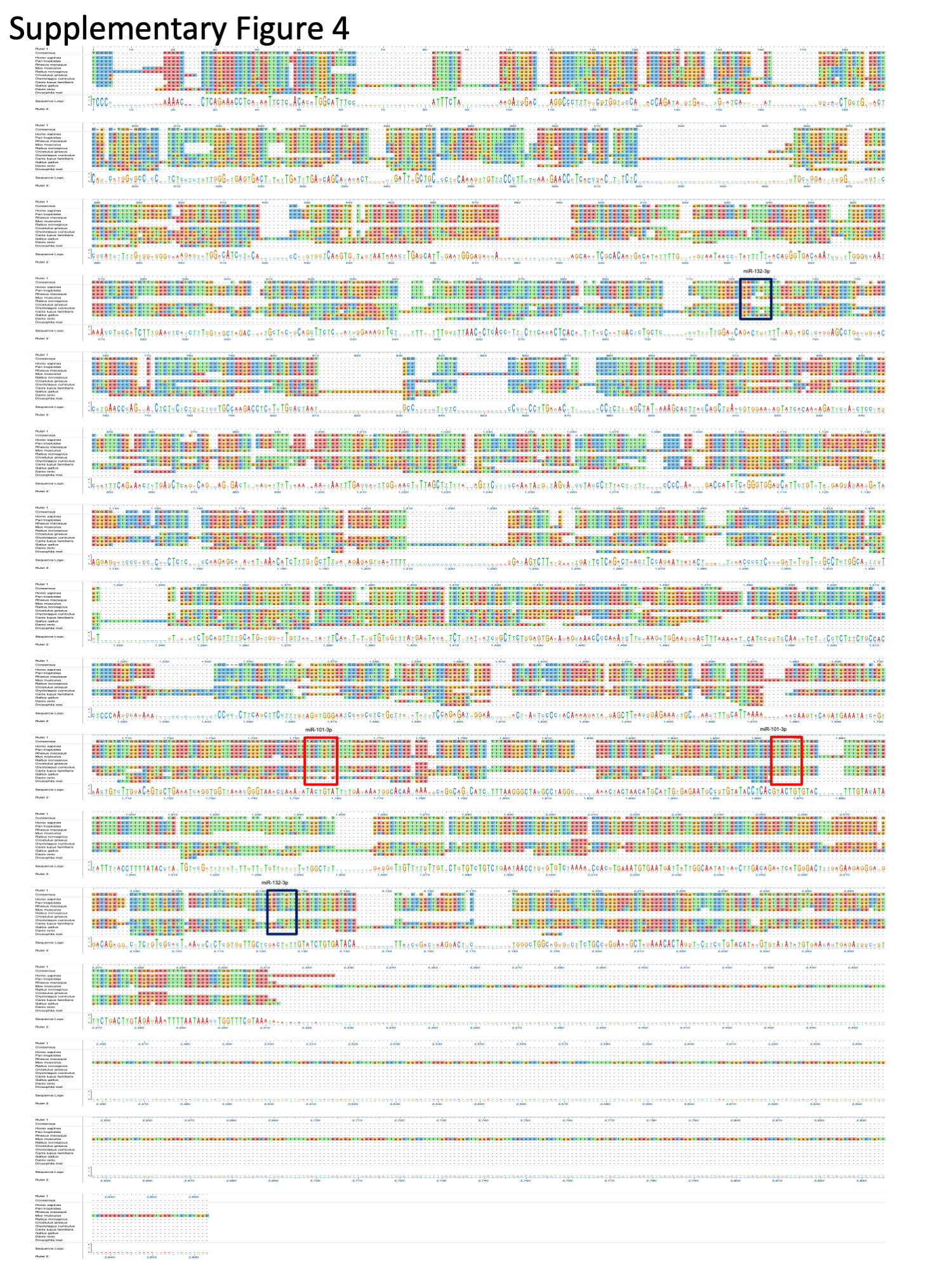

Supplement: Supplementary Figure 4 — TRIB1 3’UTR mega alignment. Overview of TRIB1 3’UTR sequences alignment in different species, generated with the software DNASTAR Lasergene (v16); miR-101-3p and miR-132-3p binding sites are highlighted in red and black, respectively. [file Image_4.tiff]

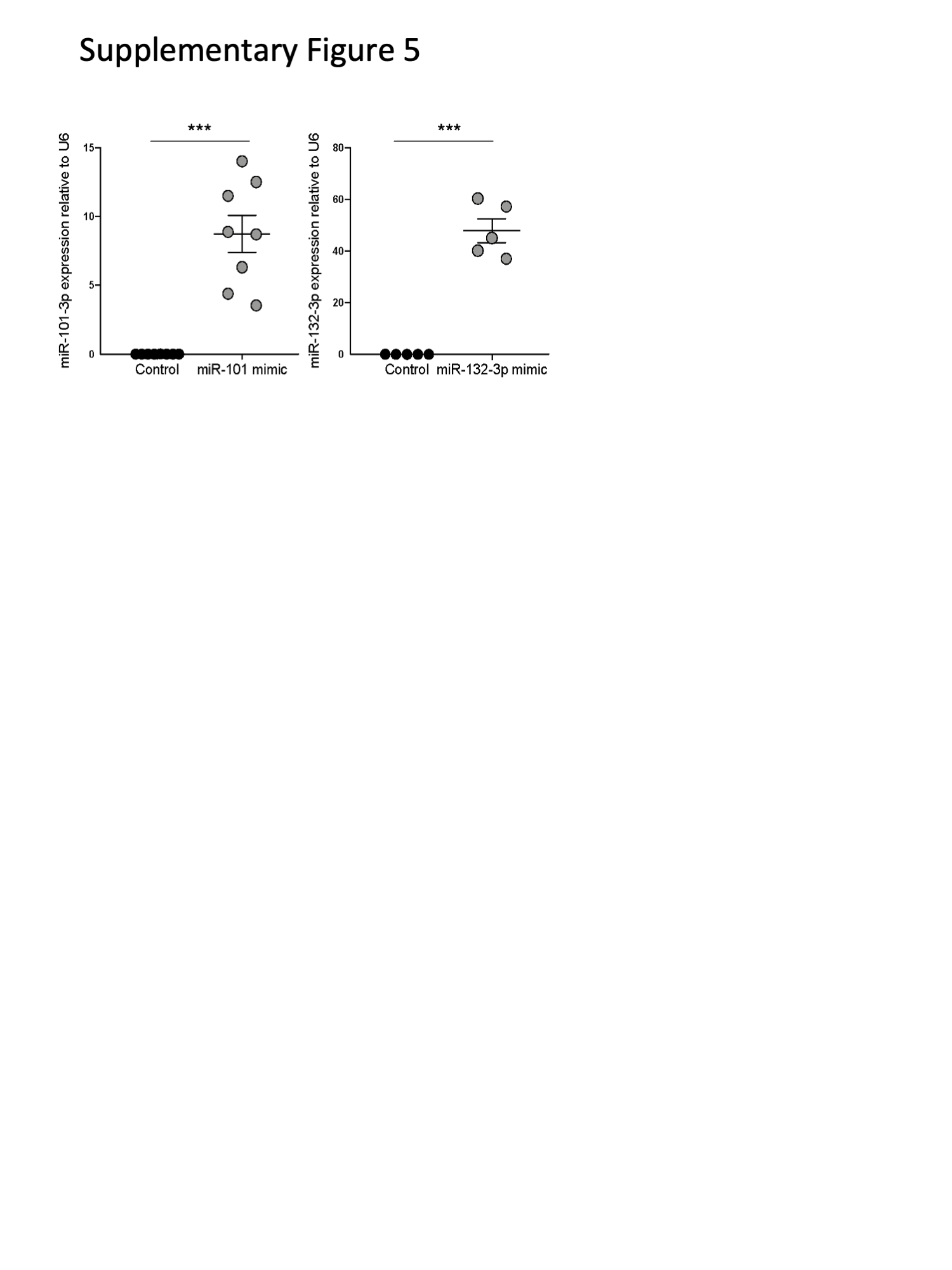

Supplement: Supplementary Figure 5 — Confirmation of miRNAs overexpression in human macrophages. Relative expression of miR-101-3p and miR-132-3p normalized to the housekeeping U6 in MDMs transiently transfected with miRNA mimics (n = 5–8, biological replicates, paired t-test, ***p < 0.001). Data are presented as mean ± SEM (scatter dot plot where each dot represents an individual donor of MDMs). Biological replicates represent the average of 3 technical replicates. Transfection was carried out for 24 h, employing 50nM of mimics and negative control. RT-qPCR data were analyzed using the 2(−Δct) method. [file Image_5.tiff]

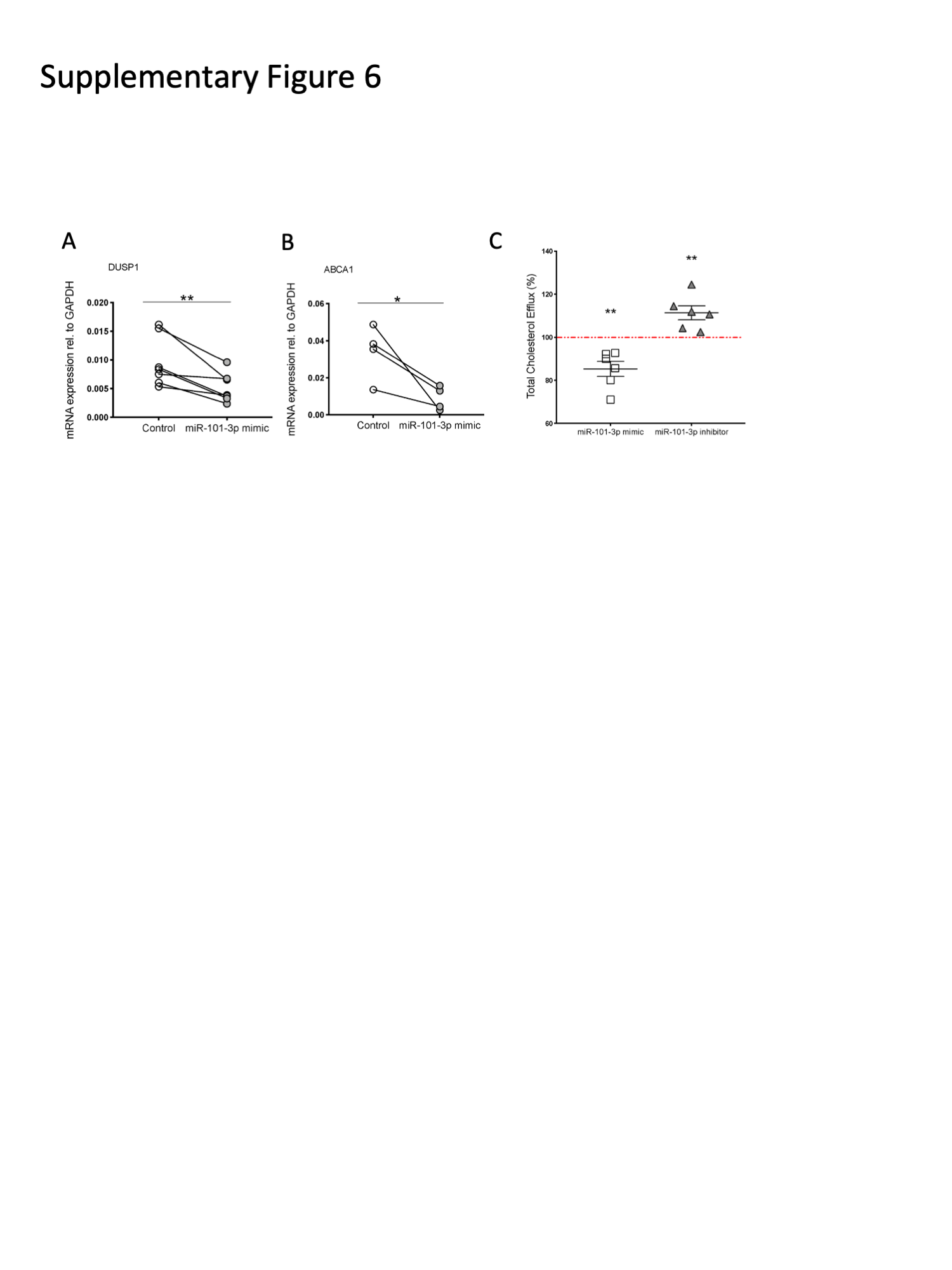

Supplement: Supplementary Figure 6 — Effect of miR-101-3p on previously validated targets: DUSP1 and ABCA1. (A, B) Relative mRNA expression of DUSP1 and ABCA1 normalized to the housekeeping GAPDH in MDMs transiently transfected with miR-101-3p mimic and a negative control (n = 4–7, biological replicates, paired t-test, **p < 0.01, *p < 0.05). (C) Percentage of total cholesterol efflux to HDL measured in MDMs transiently transfected with miR-101-3p mimic and inhibitor (n = 6, biological replicates, paired t-test, **p < 0.01). Data are presented as individual points (A, B) and as mean ± SEM (scatter dot plot) (C). Each dot represents an individual donor of MDMs; biological replicates represent the average of 3 technical replicates. Transfection was carried out for 24 h, employing 50 nM of mimic/control and 25 nM of inhibitor/control. RT-qPCR data were analyzed using the 2(−Δct) method. As miRNA mimic and inhibitor were used at different concentrations and along with their controls (negative control mimic and negative control inhibitor) we used the paired t-test. [file Image_6.tiff]
