# Supplementary material for: Tribbles-1 Expression and Its Function to Control Inflammatory Cytokines, Including Interleukin-8 Levels are Regulated by miRNAs in Macrophages and Prostate Cancer Cells
Source: Front Immunol. 2020 Nov 27;11:574046. doi: 10.3389/fimmu.2020.574046 (PMC7728618; doi:10.3389/fimmu.2020.574046)
Supplement: Supplementary Table 1 — List of RNA constructs used for transient transfection. [file Table_1.pdf]

Supplementary Table 1

RNA	Cat. Number	Sequence
miR-101-3p mimic	C-300518-07-0005	UACAGUACUGUGAUAACUGAA
miR-132-3p mimic	C-300599-06-0005	UAACAGUCUACAGCCAUGGUCG
miR-214-5p mimic	C-301153-01-0005	UGCCUGUCUACACUUGCUGUGC
miR-101-3p inhibitor	IH-300518-08-0005	NA
miR-132-3p inhibitor	IH-300599-05-0005	NA
Negative control mimic	CN-001000-01-05	NA
Negative control inhibitor	IN-001005-01-05	NA
TRIB1 siRNA SMARTpool	L-003633-00-0005	GCAAGGUGUUUCCCAUUAA<br>CUAGAAGACACACACAUAA<br>CGGAAAGGCUGCGGACGUU<br>GAACCCAGCUUAGACUAGA
siRNA negative control	D-001810-01-05	UGGUUUACAUGUCGACUAA
