# Supplementary material for: Tribbles-1 Expression and Its Function to Control Inflammatory Cytokines, Including Interleukin-8 Levels are Regulated by miRNAs in Macrophages and Prostate Cancer Cells
Source: Front Immunol. 2020 Nov 27;11:574046. doi: 10.3389/fimmu.2020.574046 (PMC7728618; doi:10.3389/fimmu.2020.574046)
Supplement: Supplementary Table 2 — List of primary and secondary antibodies used for western blot (WB) and immunofluorescence (IF) experiments. [file Table_2.pdf]

Supplementary Table 2

Antibody	Company and Cat. Number	Working Dilution	Incubation
Trib1	#09-126, Millipore	1:1000 WB	O/N, 4℃
Alpha-tubulin	sc-5286, Santa Cruz Biotech	1:5000 WB	O/N, 4℃
Heat Shock Protein 90	ab13495, Abcam	1:5000 WB	O/N, 4℃
Anti-Mouse	P0447, Dako	1:5000 WB	1hr, RT
Anti-Rabbit	P0448, Dako	1:5000 WB	1hr, RT
Rat anti-F4/80	#565409 (Clone T-45-2342), BD Pharmingen	1:50 IF	1hr, RT
Biotinylated rabbit anti-rat	Vector Laboratories, UK	1:200 IF	30 mins, RT
PE-Streptavidin	#405203, BioLegend	1:20 IF	30 mins, RT
Ym1	Anti-Chitinase like-protein 3 #ab93034, Abcam	1:50 IF	1hr, RT
IRF5	Interferon regulatory factor-5, #ab33478, Abcam	1:50 IF	1hr, RT
Donkey anti-rabbit NL493	#NL006, R&D systems	1:100 IF	1hr RT
Donkey anti-mouse NL493	#NL009, R&D systems	1:100 IF	1hr RT
