# Supplementary material for: Tribbles-1 Expression and Its Function to Control Inflammatory Cytokines, Including Interleukin-8 Levels are Regulated by miRNAs in Macrophages and Prostate Cancer Cells
Source: Front Immunol. 2020 Nov 27;11:574046. doi: 10.3389/fimmu.2020.574046 (PMC7728618; doi:10.3389/fimmu.2020.574046)
Supplement: Supplementary Table 3 — List of RT-qPCR primers. [file Table_3.pdf]

Supplementary Table 3

Gene	Forward primer	Reverse primer
hTRIB1	CTCCACGGAGGAGAGAACCC	GACAAAGCATCATCTTCCCCC
hGAPDH	ATTGCCCTCAACGACCACTTT	CCCTGTTGCTGTAGCCAAATTC
hIL-4	TCCGATTCCTGAAACGGCTC	TGGTTGGCTTCCTTCACAGG
hIL-6	ACCCCCAGGAGAAGATTCCA	GATGCCGTCGAGGATGTACC
hIL-8	TGCCAAGGAGTGCTAAAG	CTCCACAACCCTCTGCAC
hMSR-1	CGAGGTCCCACTGGAGAAAGT	CAATTGCTCCCCGATCACCTTT
hCD36	TCTGTCCTATTGGGAAAGTCACTG	GAACTGCAATACCTGGCTTTTCTC
hCD163	AGGAGAGAACTTAGTCCACCA	TCAGAATGGCCTCCTTTTCCA
hSPAR-C	TGATGGTGCAGAGGAAACCG	TGTTCTCATCCAGCTCGCAC
hPD-L1	AGGGCATTCCAGAAAGATGAGG	GGTCCTTGGGAACCGTGAC
hTNF-α	CCTGCTGCACTTTGGAGTGA	CTTGTCACTCGGGGTTCGAG
hCD80	TGCCTGACCTACTGCTTTGC	GGCGTACACTTTCCCTTCTCA
hCD86	CCCAGACCACATTCCTTGGAT	TCCCTCTCCATTGTGTTGGT
hABCA1	TACATCTCCCTTCCCGAGCA	GGGCCAGAGTCCCAAGACTA
hDUSP1	CCCCACTCTACGATCAGGGT	CCTTGCGGGAAGCGTGATA
mTrib1	CTTACATCCAGCTGCCGTCC	GTAGGCCTTGCTCTCACCAA
mGapdh	TGGCAAAGTGGAGATTGTTGCC	AAGATGGTGATGGGCTTCCCG
