# Supplementary material for: Tribbles-1 Expression and Its Function to Control Inflammatory Cytokines, Including Interleukin-8 Levels are Regulated by miRNAs in Macrophages and Prostate Cancer Cells
Source: Front Immunol. 2020 Nov 27;11:574046. doi: 10.3389/fimmu.2020.574046 (PMC7728618; doi:10.3389/fimmu.2020.574046)
Supplement: Supplementary Table 4 — List of PCR primers used for site-directed mutagenesis. [file Table_4.pdf]

Supplementary Table 4

Site-directed mutagenesis primers	Sequence
del1526-1532	GAGAATGCCGTGTATACCTCACGTACTTTGTACATATATTTTACC
del1526-1532-antisense	GGTAAAATATATGTACAAAGTACGTGAGGTATACACGGCATTCTC
