# Supplementary material for: Tribbles-1 Expression and Its Function to Control Inflammatory Cytokines, Including Interleukin-8 Levels are Regulated by miRNAs in Macrophages and Prostate Cancer Cells
Source: Front Immunol. 2020 Nov 27;11:574046. doi: 10.3389/fimmu.2020.574046 (PMC7728618; doi:10.3389/fimmu.2020.574046)
Supplement: Supplementary Table 5 — List of “high-confidence” miRNAs predicted to target the 3’UTR of TRIB1 by 3 different prediction tools, along with their features taken from TargetScan. The list of “high-confidence” miRNAs can be accessed and downloaded using this link ftp://mirbase.org/pub/mirbase/CURRENT (miRbase v.22). [file Table_5.pdf]

Supplementary Table 5

High-Confidence miRNAs	Target site position	Seed region	No. of prediction tools
hsa-let-7a-5p	1518-1524	7mer-1A	6
hsa-let-7b-5p	1518-1524	7mer-1A	6
hsa-let-7d-5p	1518-1524	7mer-1A	5
hsa-miR-101-3p	1526-1532, 1424-1430	7mer-m8, 7mer-1A	7
hsa-miR-10a-5p	391-397	7mer-m8	4
hsa-miR-10b-5p	391-397	7mer-m8	4
hsa-miR-125a-5p	862-868	7mer-m8	4
hsa-miR-125b-5p	862-868	7mer-m8	4
hsa-miR-1287-5p	1259-1265	7mer-m8	3
hsa-miR-129-1-3p	1467-1474	8mer	5
hsa-miR-129-2-3p	1467-1474	8mer	5
hsa-miR-132-3p	554-560, 1763-1769	7mer-m8, 7mer-m8	6
hsa-miR-136-5p	1321-1328	8mer	3
hsa-miR-144-3p	1423-1430	8mer	6
hsa-miR-150-5p	250-256, 336-342	7mer-m8, 7mer-1A	3
hsa-miR-154-5p	1635-1641	7mer-m8	4
hsa-miR-2116-3p	335-342	8mer	3
hsa-miR-22-3p	458-464	7mer-m8	3
hsa-miR-23a-3p	1662-1669, 1869-1875	8mer, 7mer-1A	6
hsa-miR-302a-3p	680-687	8mer	6
hsa-miR-302c-3p	680-687, 679-685	8mer, 7mer-m8	6
hsa-miR-328-3p	406-412	7mer-1A	3
hsa-miR-330-3p	947-954, 1599-1605	8mer, 7mer-1A	5
hsa-miR-330-5p	1281-1287	7mer-1A	4
hsa-miR-382-3p	1667-1673	7mer-m8	4
hsa-miR-485-5p	1262-1268	7mer-m8	3
hsa-miR-548o-3p	1060-1066	7mer-m8	4
hsa-miR-574-5p	496-502, 512-519	7mer-1A, 8mer	6
hsa-miR-616-3p	357-364, 527-533	8mer, 7mer-m8	5
hsa-miR-625-5p	661-667	7mer-m8	4
hsa-miR-642a-5p	1715-1721	7mer-m8	3
hsa-miR-654-3p	928-934	7mer-m8	3
hsa-miR-873-5p	611-617	7mer-m8	4
hsa-miR-876-5p	1225-1231	7mer-m8	3
hsa-miR-942-5p	885-892, 1711-1717	8mer, 7mer-1A	4
