# Supplementary material for: Tribbles-1 Expression and Its Function to Control Inflammatory Cytokines, Including Interleukin-8 Levels are Regulated by miRNAs in Macrophages and Prostate Cancer Cells
Source: Front Immunol. 2020 Nov 27;11:574046. doi: 10.3389/fimmu.2020.574046 (PMC7728618; doi:10.3389/fimmu.2020.574046)
Supplement: Supplementary Table 6 — The table lists candidate miRNAs selected for experimental validation and their features, according to TargetScan; both were listed as “high-confidence” miRNAs, according to deep sequencing published data (miRbase v.22). [file Table_6.pdf]

Supplementary Table 6

Candidate miRNA	High Confidence (miRbase v.22)	Target site position and seed region type	Phylogenetic conservation	Number of prediction tools	Relevant publications (macrophage, inflammation)
miR-101-3p	Yes	Position 1526-1532, 7mer m8	++++	7	40, 41
		Position 1424-1430, 7mer A1	+	2	
miR-132-3p	Yes	Position 554-560, 7mer m8	+	3	63, 64, 65
		Position 1763-1769, 7mer m8	+	6	
