# Supplementary material for: Tribbles-1 Expression and Its Function to Control Inflammatory Cytokines, Including Interleukin-8 Levels are Regulated by miRNAs in Macrophages and Prostate Cancer Cells
Source: Front Immunol. 2020 Nov 27;11:574046. doi: 10.3389/fimmu.2020.574046 (PMC7728618; doi:10.3389/fimmu.2020.574046)
Supplement: Supplementary Table 7 — List of miRNAs downregulated/silenced in prostate cancer and predicted to target the 3’UTR of TRIB1. miRNA-target prediction analysis was performed by using TargetScan, miRanda and Starbase. The list of dysregulated miRNAs in prostate cancer was taken from miRCancer and it is accessible at this link: http://mircancer.ecu.edu/search.jsp?mirId=&logic=&condition=Or&cancerName=prostate+cancer&buttonSearch. [file Table_7.pdf]

Supplementary Table 7

miRNAs	No. of predicted binding sites	Seed region type	Status in PCa	PCa publications
hsa-let-7a-3/5p	3	7mer	Downregulated	66, 67
hsa-let-7c-3/5p	2	7mer	Downregulated	68
hsa-miR-101-3p	2	7mer	Downregulated	69, 70, 71
hsa-miR-10a-5p	1	7mer	Downregulated	72
hsa-miR-129-3p	1	8mer	Downregulated	73, 74
hsa-miR-130a-3/5p	2	7mer, 8mer	Downregulated	75
hsa-miR-132-3p	2	7mer	Downregulated	38, 39,
hsa-miR-144-3p	1	8mer	Downregulated	76
hsa-miR-150-3/5p	3	7mer	Downregulated	77
hsa-miR-154-5p	2	7mer	Downregulated	78
hsa-miR-212-3p	2	7mer	Downregulated	79, 80
hsa-miR-22-3p	1	7mer	Downregulated	81
hsa-miR-224-5p	1	7mer	Downregulated	47, 82, 83
hsa-miR-23a-3/5p	3	7mer, 8mer	Downregulated	84
hsa-miR-23b-3/5p	2	7mer, 8mer	Downregulated	85
hsa-miR-302a-3p	1	8mer	Downregulated	86
hsa-miR-330-3/5p	2	7mer, 8mer	Downregulated	87, 88
hsa-miR-3619-3/5p	2	7mer	Downregulated	89
hsa-miR-372-3/5p	2	7mer, 8mer	Downregulated	90
hsa-miR-373-3p	1	8mer	Downregulated	91
hsa-miR-382	1	7mer	Downregulated	92
